# Tuning of MoS_2_ Photoluminescence in Heterostructures with CrSBr

**DOI:** 10.1021/acsami.5c01924

**Published:** 2025-04-15

**Authors:** Satyam Sahu, Oleksandr Volochanskyi, Vaibhav Varade, Luka Pirker, Viktor Zólyomi, János Koltai, Kseniia Mosina, Zdeněk Sofer, Otakar Frank, Jana Vejpravová, Martin Kalbáč, Matěj Velický

**Affiliations:** † J. Heyrovský Institute of Physical Chemistry, Czech Academy of Sciences, Dolejškova 2155/3, Prague 182 23, Czech Republic; ‡ Department of Biophysics, Chemical and Macromolecular Physics, Faculty of Mathematics and Physics, Charles University, Ke Karlovu 3, Prague 121 16, Czech Republic; § Department of Physical Chemistry, Faculty of Chemical Engineering, University of Chemistry and Technology in Prague, Technická 5, Prague 142 00, Czech Republic; ∥ Department of Condensed Matter Physics, Faculty of Mathematics and Physics, Charles University, Ke Karlovu 5, Prague 121 16, Czech Republic; ⊥ Hartree Centre, STFC Daresbury Laboratory, Daresbury WA4 4AD, U.K.; # Department of Biological Physics, Eötvös Loránd University, Pázmány Péter sétány 1/A, Budapest 1117, Hungary; ¶ Department of Inorganic Chemistry, University of Chemistry and Technology Prague, Technická 5, 166 28 Prague, Czech Republic

**Keywords:** MoS_2_, CrSBr, heterostructures, photoluminescence, enhancement, quenching, optoelectronics

## Abstract

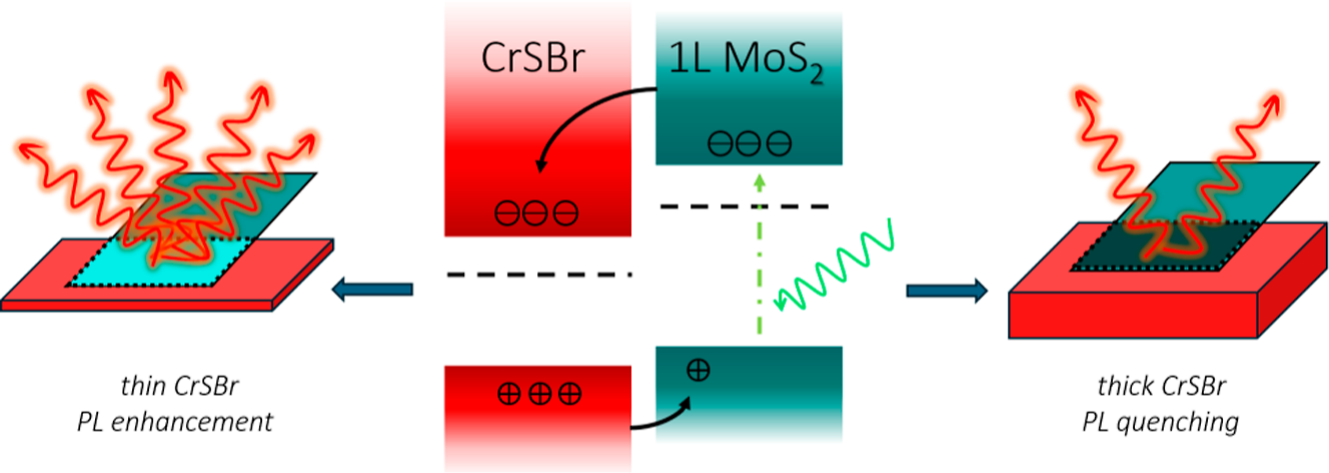

Monolayers of semiconducting transition metal dichalcogenides (TMDCs) are known for their unique excitonic photoluminescence (PL), which can be tuned by interfacing them with other materials. However, integrating TMDCs into van der Waals heterostructures often results in a significant quenching of the PL because of an increased rate of nonradiative recombination processes. We demonstrate a wide-range tuning of the PL intensity of monolayer MoS_2_ interfaced with another layered semiconductor, CrSBr. We discover that a thin CrSBr up to ≈20 nm in thickness enhances the PL of MoS_2_, while a thicker material causes PL quenching, which is associated with changes in the excitonic makeup driven by the charge redistribution in the CrSBr/MoS_2_ heterostructure. Transport measurements, Kelvin probe force microscopy, and first-principles calculations indicate that this charge redistribution most likely causes n- to p-type doping transition of MoS_2_ upon contact with CrSBr, facilitated by the type II band alignment and the tendency of CrSBr to act as an electron sink. Furthermore, we fabricate an efficient AC-regime photodetector with a responsivity of 10^5^ A/W from a MoS_2_/CrSBr heterostructure.

## Introduction

Recently, two-dimensional (2D) materials have become a focal point for scientists in many disciplines due to their unique mechanical, electrical, and optical properties, when thinned down to a few atomic layers.^1−6^ Among these materials, transition metal dichalcogenides (TMDCs) have emerged as particularly exciting due to the direct band gap transition in their monolayers (1L),^5,6^ which makes them ideal for optoelectronic applications. 1L TMDCs exhibit large binding energies of neutral excitons (A^0^, B^0^) and negatively (A^–^) or positively (A^+^) charged trions, valley selection, and strong spin–orbit coupling.^7−10^ Due to these exotic properties and thickness-dependent tunable band gaps, ranging from the visible to near-infrared spectrum, TMDCs are highly considered for next-generation applications.^2,11,12^

Recently, van der Waals (vdW) heterostructures have emerged as promising artificial systems for enhancing and tailoring the optical and electrical properties of 2D materials. These heterostructures, composed of atomically thin layers stacked on top of each other, offer unique opportunities to engineer novel functionalities by combining different materials with suitable properties.^13,14^ The key aspect of semiconducting vdW heterostructures is the type of band alignment of the two constituent materials, which influences the optoelectronic properties of the composite system.^15−18^

In a type I band alignment, the conduction band minimum (CBM) and valence band maximum (VBM) are both located in the 2D layer with the smaller band gap, facilitating efficient charge transfer from the larger band gap material to the smaller band gap material, thus enhancing the photoluminescence (PL) intensity of the latter.^15,18^ In contrast, type II band alignment features a staggered gap with CBM and VBM in different 2D layers. Type II alignment leads to spatially separated charge carriers, extended carrier lifetimes, and tunable emission wavelengths.^16^ In most cases, the PL of the larger band gap material tends to be suppressed for both band alignments, whereas the PL of the smaller band gap material is either enhanced (type I) or quenched (type II).^19^ However, PL enhancement of the larger band gap material has also been observed due to charge depletion.^16^ Crucially, either the enhancement or the quenching, but not both, can usually be observed for a particular combination of materials without any external perturbation.^15,16,20^

We demonstrate PL tuning of 1L MoS_2_ via thickness modulation of an adjacent CrSBr crystal in a type II vdW heterostructure, governed by the charge carrier transfer. Both 1L MoS_2_ and CrSBr (all thicknesses) are direct band gap semiconductors with an optical band gap of 1.88^6^ and 1.36 eV,^21,22^ respectively. We achieved neutral exciton modulation in 1L MoS_2_ by a factor of 0.5 to 16. The overall PL modulation varied by a factor of 0.1 to 10 through carrier extraction from MoS_2_, providing a versatile platform for tailoring light emission properties. Notably, we demonstrate fine-tuning of PL, spanning both enhancement and quenching regimes, underscoring the potential of vdW heterostructures for next-generation optoelectronic devices, as exemplified by a MoS_2_/CrSBr photodetector with a responsivity of 10^5^ A/W, operating in the AC regime.

## Results and Discussion

### Enhancement and Quenching of MoS_2_ Photoluminescence

**Figure 1:**
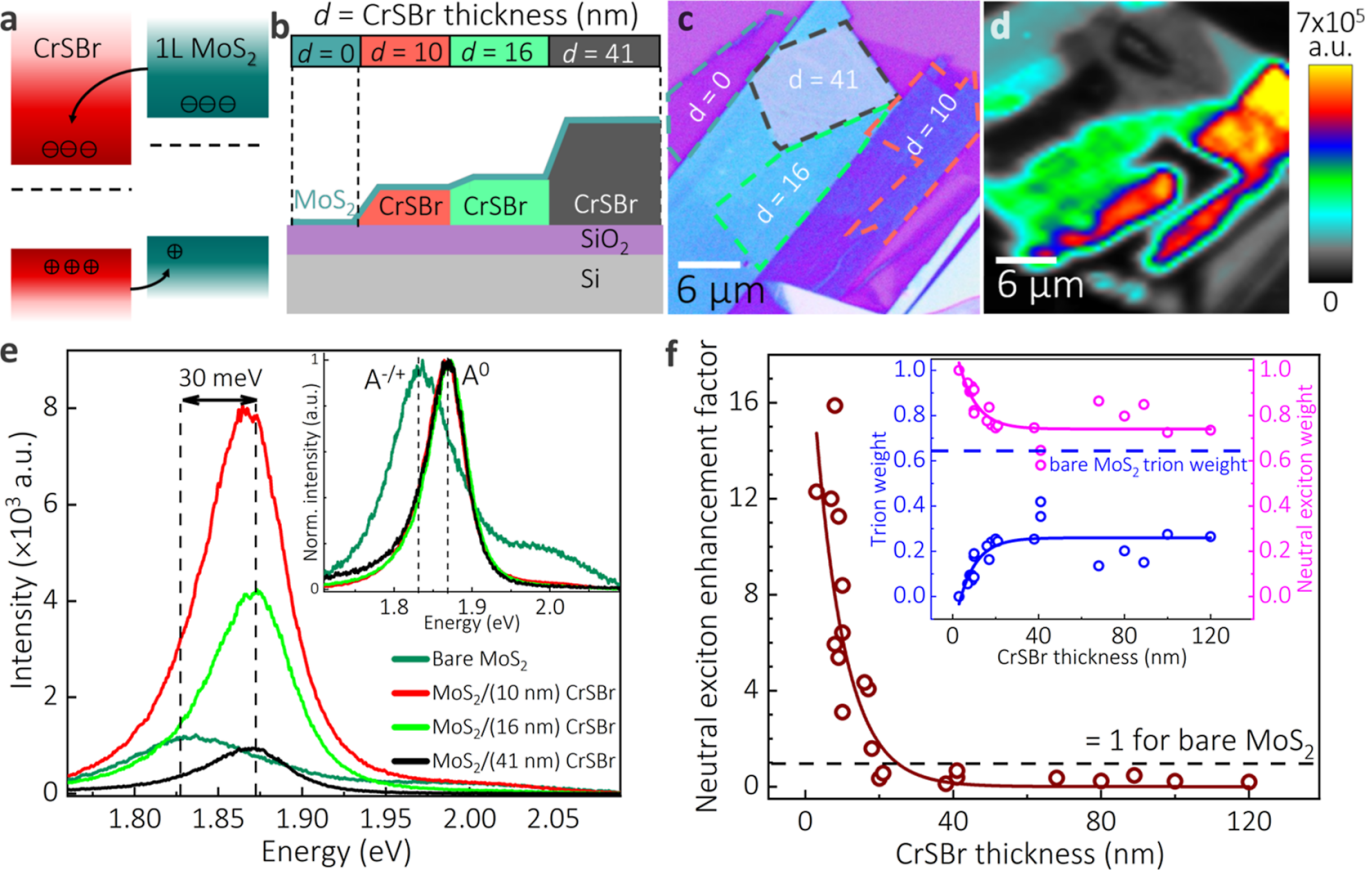
Enhancement and quenching of monolayer MoS_2_ photoluminescence in MoS_2_/CrSBr heterostructure. (a) Schematic of the predicted type II band alignment of 1L MoS_2_/CrSBr and the charge transfer processes. (b) Schematic of the MoS_2_/CrSBr heterostructure cross-section with the colored bar showing the different thicknesses of CrSBr. (c) Optical image of a representative sample with different regions marked. (d) Room-temperature spatial map of the 1L MoS_2_ PL intensity for the 1.65–2.05 eV spectral region of the same sample at 2.33 eV excitation. (e) Averaged PL spectra of the four different regions. Inset: PL spectra normalized to the maximum intensity of each spectrum. (f) Neutral exciton PL enhancement factor as a function of the CrSBr thickness. The horizontal dashed line corresponds to the PL of bare MoS_2_ on SiO_2_ (enhancement factor = 1). Inset: trion and neutral exciton weights as a function of the CrSBr thickness. The horizontal dashed line indicates the trion weight for the bare MoS_2_ on SiO_2_. The solid curves are guides for the eye. The enhancement factors and trion/exciton weights were estimated from single spectra for different CrSBr thicknesses.

Figure 1a shows a schematic of the type II band alignment for MoS_2_/CrSBr heterostructure predicted by first-principles calculations and confirmed by Kelvin probe force microscopy (KPFM) measurements (Supporting Information Sections S1–S3). Figure 1b represents a cross-section of one of the heterostructures measured in this study, depicting CrSBr flakes of different thicknesses (*d*) covered by 1L MoS_2_ (thickness estimation in Supporting Information Section S4). In all the heterostructures, MoS_2_ was transferred on top of CrSBr with a part of the MoS_2_ flake remaining in direct contact with the SiO_2_/Si substrate (left-most region in Figure 1b), as a reference. A representative optical image of the sample is shown in Figure 1c. The colored dashed borders outline the various heterostructure regions, corresponding to the color scheme in Figure 1b, and to the spatial map of the 1L MoS_2_ PL intensity (integrated peak area) in Figure 1d.

The different heterostructure regions include bare MoS_2_ on SiO_2_ (teal), MoS_2_ with quenched PL on 41 nm-thick CrSBr (gray), and MoS_2_ on 10- and 16 nm CrSBr representing regions of high (red-yellow) and moderate (green) PL enhancement of MoS_2_, respectively. The PL map of the spectral region (1.65–2.05 eV) is reasonably uniform with variation in intensity <10% within the respective regions, suggesting homogeneous PL modulation. Next, we compare the averaged PL spectra of MoS_2_ from all four regions in Figure 1e. We observe a broad emission in the range of 1.82–1.87 eV in the PL spectrum of bare MoS_2_ on SiO_2_, corresponding to A^-/+^ and A^0^, along with B^0^ at 2.02 eV.^5,23^ These emission peaks arise from the recombination of excitons, originating from the electrons and holes at the K and K′ points of the band structure.^5,6^ In contrast, there is a strong single peak at 1.87 eV corresponding to A^0^, with a slight asymmetry at around 1.83 eV attributed to A^-/+^, and no B^0^ emission, for all three MoS_2_/CrSBr heterostructure regions (see Supporting Information Section S5 for fitted spectra). This suggests strong quenching of A^-/+^ and complete extinction of B^0^ in the heterostructure regions (inset of Figure 1e), as a result of the charge transfer in the predicted type II band alignment shown in Figure 1a.

Crucially, the PL intensity of 1L MoS_2_ varies for different thicknesses of CrSBr. The PL emission associated with the A exciton coming from the heterostructure is brighter and narrower than on bare MoS_2_, with an intensity about 8 (≈ 4) times higher for MoS_2_ on 10 nm (16 nm) CrSBr, respectively. The emission is quenched but still narrowed for MoS_2_ on 41 nm CrSBr. To understand these pronounced changes in the PL intensity, we systematically varied the thickness of the CrSBr flake and measured the PL spectra of MoS_2_. We then decomposed the PL peak arising from the A exciton into the two components, A^0^ and A^-/+^, and plotted their spectral weights (weight of species *X* = Area of *X*/Total Area) in the inset of Figure 1f. For bare MoS_2_ on SiO_2_/Si, the A^-/+^ contribution at 1.83 eV outweighs that of A^0^ at 1.87 eV due to the typical n-doping in MoS_2_ on SiO_2_ accompanied by the presence of a negatively charged A^–^ trion.^23,24^

Upon bringing MoS_2_ in contact with the thinnest (∼3 nm) CrSBr, the A^–^ contribution vanishes due to a complete depletion of the excess electrons from MoS_2_. Since the neutral A^0^ exciton has roughly a 10-fold larger binding energy than the negative A^–^ trion,^7,25^ the electron–hole recombination rate and, therefore, the A^0^ emission are enhanced. This process is associated with the Fermi level (*E*_F_) shift from the n-type toward the undoped state. The trion contribution then starts to increase again with an increase in the CrSBr thickness (inset of Figure 1f), manifesting as PL quenching. However, this time it is most likely associated with the positive A^+^ trion due to the further boost in electron transfer from MoS_2_ to CrSBr and/or holes from CrSBr to MoS_2_.^15^ This hypothesis asserts that the increasing thickness of CrSBr makes it a more efficient electron sink for MoS_2_, facilitating the n- to p-type charge doping transition in MoS_2_.

From the A^0^ contributions to the PL of MoS_2_, we estimate the neutral exciton enhancement factor (ratio of the A^0^ intensity on CrSBr to that on SiO_2_) and plot it as the function of the CrSBr thickness (Figure 1f). The enhancement factor decreases with increasing CrSBr thickness, reaching a unity, that is, the threshold between enhancement and quenching, at approximately 20 nm. Beyond this threshold, the PL intensity drops to roughly half that observed for MoS_2_ on SiO_2_ and remains stable for thicker CrSBr flakes. The MoS_2_/CrSBr material combination is unique for its ability to both enhance and quench the MoS_2_ PL, a significant advancement over previous studies that achieved only one of the effects.^15,16,20^

### n- to p- Doping Transition in MoS_2_

**Figure 2:**
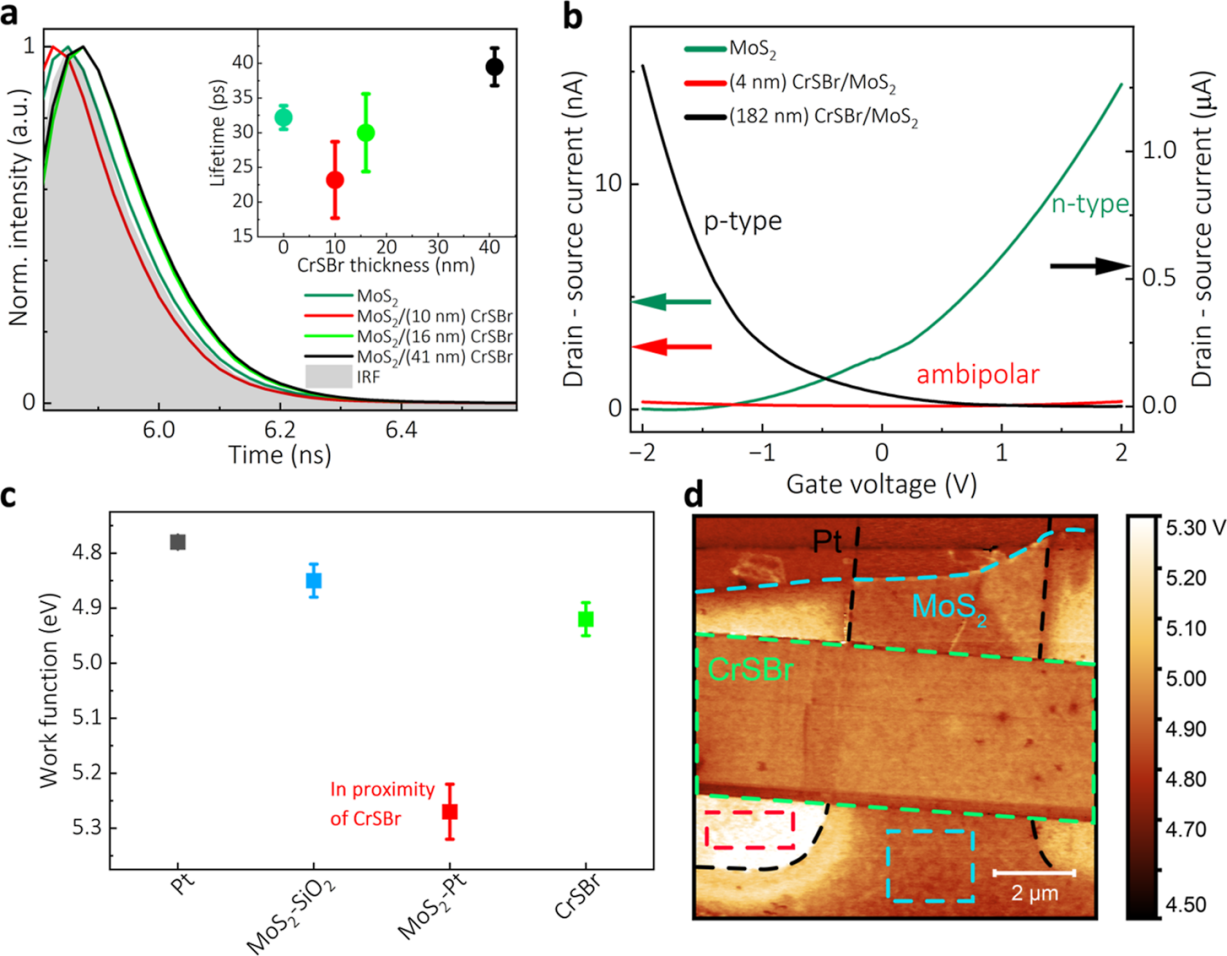
n- to p- doping transition in MoS_2_. (a) Time-resolved PL for the different heterostructure regions. Gray shaded region is the instrument response function. Inset: Average radiative exciton lifetimes for different regions with the standard deviations for at least 20 measurements in each region. (b) Transfer curves for devices with bare MoS_2_ (teal), enhanced PL region (red; 4 nm CrSBr), and quenched PL region (black; 182 nm CrSBr), showing the n- to p- doping transition in MoS_2_ passing through the quasi-ambipolar state. Measured in the dark. (c) Work functions of the Pt electrode (gray), 1L MoS_2_ on SiO_2_ (blue), 1L MoS_2_ on Pt in proximity to CrSBr (red), and thick CrSBr (green), measured by KPFM. Note that the bending rigidity of CrSBr prevents direct contact with MoS_2_ in the channel area, where the latter sags down to the SiO_2_ substrate (Supporting Information Section S6). (d) KPFM map of thick CrSBr on top of MoS_2_. Areas marked with dashed lines were used to estimate the average work functions shown in panel c.

Figure 2a shows the normalized time-resolved PL decay for all four heterostructure regions (colored) and the instrument response function (shaded gray). The inset of Figure 2a shows the average PL lifetimes extracted from at least 20 measurements for each region. The decay time of MoS_2_ PL is caused by various factors, including the effective exciton radiative recombination time, nonradiative recombination channels, such as exciton–exciton annihilation, and trion formation.^26−28^*E*_F_ controls the dominant nonradiative recombination pathway.^29^

The variations in average exciton lifetimes across different regions are consistent with the observed PL enhancement and quenching. Bare MoS_2_ on SiO_2_, which is n-doped, has a lifetime of 32 ± 2 ps. For MoS_2_ on 10 nm CrSBr, positive A^+^ trions are present at significantly lower weights (≈ 15%) than negative A^–^ trions in bare MoS_2_ (≈ 65%), leading to a shorter lifetime, which was estimated to be below the instrumental resolution of 25 ps. MoS_2_ on 16 nm CrSBr has a higher A^+^ weight (≈ 20%) than MoS_2_ on 10 nm CrSBr, and a marginally shorter lifetime of 30 ± 2 ps. Finally, for MoS_2_ on 41 nm CrSBr, in which quenching occurs, electron depletion causes strong p-doping and the highest A^+^ trion weight (≈ 25%), extending the lifetime beyond that of bare MoS_2_ to 40 ± 3 ps.

We also performed transport measurements (Figure 2b) on three different sets of vertical heterojunctions on SiO_2_/Si: bare MoS_2_, thin (4 nm) CrSBr on top of MoS_2_, and thick (182 nm) CrSBr on top of MoS_2_ (device geometry is shown in the Supporting Information Section S6). Starting with bare MoS_2_ (teal), we observed the typical n-type behavior, confirming the presence of excess electrons. For the MoS_2_ with thin CrSBr on top (red), the behavior was ambipolar to slightly n-doped, supporting our hypothesis of excess electrons being transferred from MoS_2_ to CrSBr. Lastly, for the thick CrSBr on top (black), the MoS_2_ behavior appears strongly p-doped, suggesting that most of the MoS_2_ conduction band electrons transfer to CrSBr, leaving holes in the valence band of MoS_2_ behind.

The shifts of the *E*_F_ in the devices used for transport measurements are also seen as work function changes in KPFM measurements shown in Figure 2c,d. Work function of the Pt electrode was 4.78 ± 0.01 eV, which is lower than the UHV reported values and indicates surface contamination originating from the fabrication process.^30^ Bare MoS_2_ on SiO_2_ has a slightly higher work function of 4.85 ± 0.03 eV, which is lower than reported previously.^31^ The work function of thick CrSBr is 4.92 ± 0.03 eV. However, for MoS_2_ on the Pt electrode in the proximity of the thick CrSBr, the work function increases to 5.27 ± 0.05 eV, which provides further evidence of the electron transfer from MoS_2_ to CrSBr and the p-type behavior observed in transport measurements. Since the simple band alignment diagram does not account for such a dramatic increase in the work function, we suspect that other phenomena observed in transition metal chalcohalides, such as band gap renormalization,^32^ formation of surface insulator states,^33^ or band gap dependence on thickness (Supporting Information Section S1), can play a role.

### DC Properties and Band Alignment

**Figure 3:**
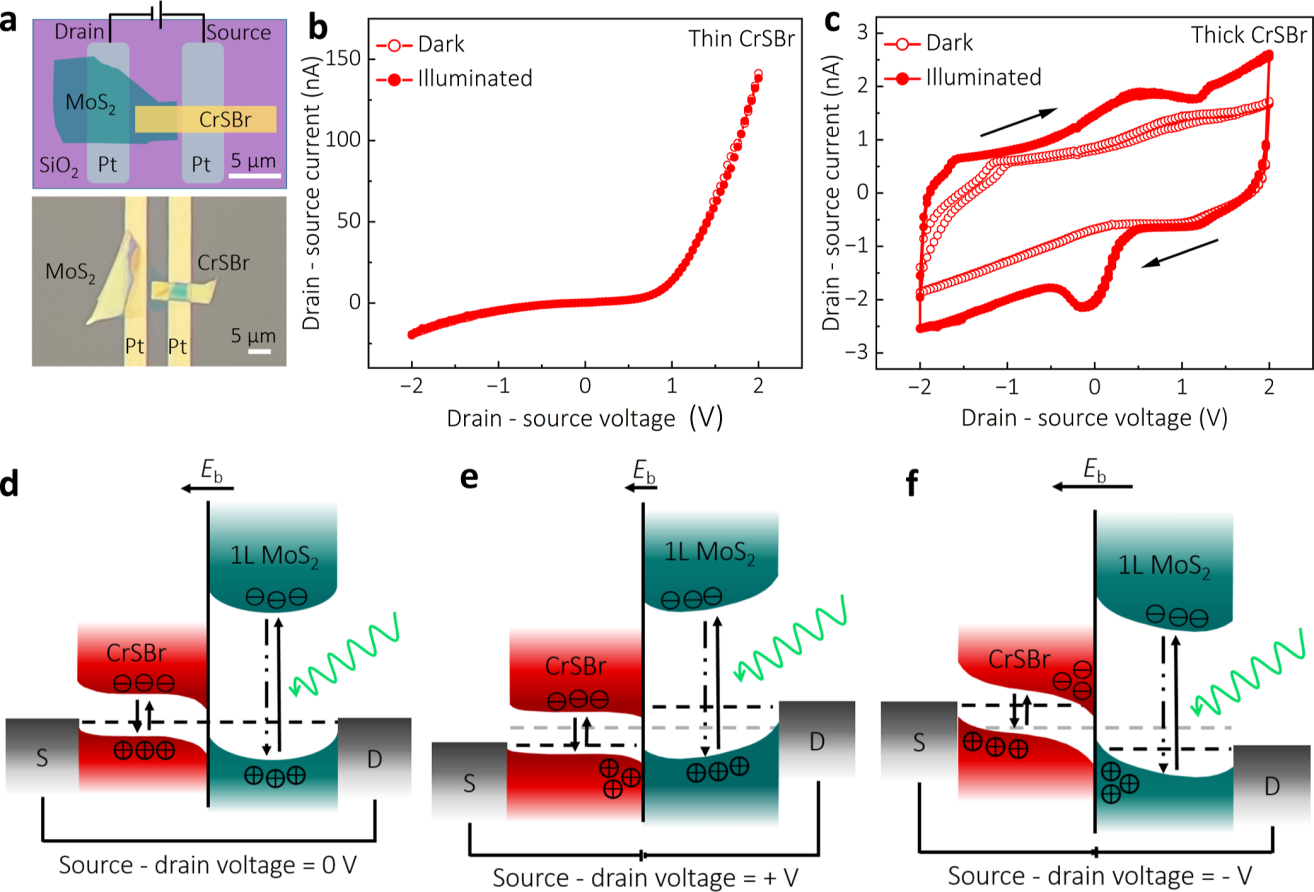
Electrical properties and band alignment of the lateral CrSBr/MoS_2_ heterojunction. (a) Schematic (top) and optical image (bottom) of the device. (b,c) Current–voltage characteristics of thin CrSBr/MoS_2_ and thick CrSBr/MoS_2_ lateral heterojunctions, respectively, in the dark (empty circles) and under white light illumination (filled circles), presented as five-cycle dual-sweep data with Pt/CrSBr as the source terminal. (d–f) Schematic energy band diagrams for a thick CrSBr/MoS_2_ heterojunction under zero, positive, and negative bias voltages, respectively (not to scale).

To further investigate the band alignment in the CrSBr/MoS_2_ heterostructure, we prepared several lateral heterojunction devices using CrSBr (MoS_2_) as the source (drain) terminal (Figure 3a). Figure 3b,c shows the current–voltage (*I*_ds_–*V*_ds_) characteristics of a typical device in the dark and under illumination for both thin (PL-enhancing) and thick (PL-quenching) CrSBr samples, respectively.

The thin CrSBr/MoS_2_ device exhibits a rectifying, diode-like response, characterized by a high output current when the CrSBr side is positively biased (Figure 3b). This behavior stems from the relatively small band bending at the heterojunction. The band alignment in this device creates conditions favorable for efficient charge recombination within MoS_2_, supported by the PL measurements and confirmed by the lack of drain-source current change upon illumination.

In contrast, the thick CrSBr/MoS_2_ device exhibits two orders of magnitude lower current due to significant band bending at the heterojunction, creating substantial barriers that hinder current flow across the device. This is evidenced by the pronounced hysteresis in *I*_ds_–*V*_ds_ observed during forward and reverse voltage sweeps (Figure 3c). An alternative explanation of the observed charging behavior involves the formation of an insulating electron crystal observed in a similar system,^33^ likely due to the high effective electron mass along the CrSBr *a*-axis.^34^ Upon photoexcitation, additional carriers are generated, increasing *I*_ds_. Notably, the current amplitude gradually builds up with each voltage step during the sweep, indicating progressive charging dynamics of the system. The opposite effect is demonstrated in the time-dependent DC photocurrent measurements under steady-state conditions using zero or finite bias voltage and interrupted illumination (Supporting Information Section S7).

The behavior of both thick and thin CrSBr devices can be attributed mainly to electrostatic charge redistribution during thermal equilibration, resulting in a built-in electric field (*E*_b_) formation directed from MoS_2_ to CrSBr (Figure 3d). In forward bias (CrSBr positively biased, Figure 3e), the built-in field is reduced, decreasing the depletion width and allowing a net current to flow through the device. In reverse bias (CrSBr negatively biased, Figure 3f), the built-in field is enhanced, expanding the depletion region and suppressing the current flow.

Given that the thickness of the CrSBr layer is the only difference between the two sample types, we can reasonably rule out the Schottky barriers at the 2D material/Pt electrode interfaces as a significant factor contributing to the difference in their *I*_ds_–*V*_ds_ curves. The presence of negative differential resistance at lower voltages during forward and reverse sweep, combined with the observed hysteresis, indicates charge trapping due to barrier height modulation and band-to-band tunneling at the heterojunction, as described in the literature.^35,36^ This suggests that, in addition to the drift current, another mechanism contributes to the operation of the thick CrSBr/MoS_2_ device.

In the lateral heterojunction device, where thick CrSBr acts as the source and MoS_2_ as the drain terminal, prominent p-type conductivity was observed (Supporting Information Section S8), similar to previous transport measurements in vertical junctions utilizing thick CrSBr layers. This highlights the consistent p-type behavior across different device configurations when using thicker CrSBr layers. Notably, the lateral junction exhibits an enhanced net hole mobility under illumination, which significantly surpasses that of electrons. Additionally, the improved transport observed upon photoexcitation may be influenced by electrons trapped in CrSBr, contributing to a photogating effect. The trapped electrons act as a negative gate, creating an electric field that repels electrons and attracts holes while shifting the *E*_F_ further downward and reinforcing strong p-type conduction, as discussed in the context of photogating of the CrSBr/MoS_2_ heterostructure in the next section. We note that defect states, such as negatively charged Br vacancies, may also contribute to photogating.^37^

### Optoelectronic Response of CrSBr/MoS_2_

In a type II band alignment of a p*-*n anisotype junction, photogenerated electron–hole pairs are separated by the offset between the CBM and VBM of the two materials. In a n-n isotype junction, however, these offsets and the static band bending constrain the DC current flow (Supporting Information Section S7), with the changes in *E*_F_ governed by the applied voltage.^38,39^ In contrast, under the AC photoexcitation, dynamic modulation of the band structure lowers the effective impedance of the device.

With the increasing AC frequency, the rapid electric field oscillations force the effective potential barrier heights to fluctuate. Charge carriers encounter an oscillating potential landscape, enabling easier charge transfer in a time-averaged manner.^40−42^ This aligns with the frequency-dependent AC characteristics of the heterojunction (Supporting Information Section S9). Current measurements in the 0.1–100 kHz range reveal negligible photocurrent generation at lower frequencies due to the dominant capacitive behavior of the interface. However, as the frequency increases, the interfacial polarization effect diminishes, enhancing photocurrent generation and interlayer charge transfer (Supporting Information Sections S10 and S11).

**Figure 4:**
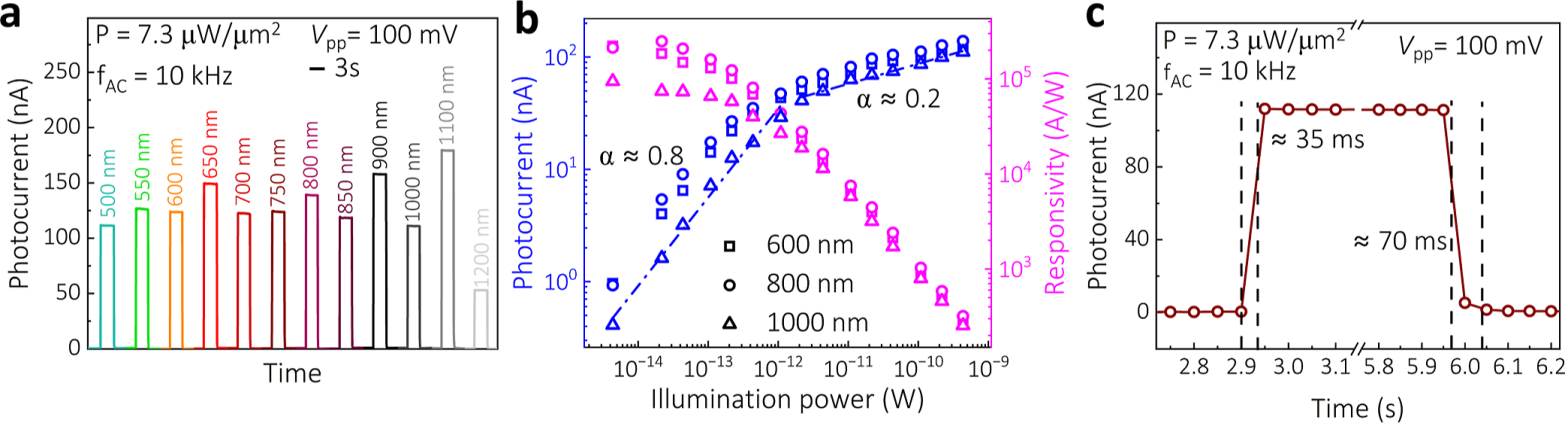
Optoelectronic operation of the CrSBr/MoS_2_ heterostructure. (a) Wavelength-dependent AC photocurrent in a thick CrSBr/MoS_2_ heterostructure measured in the spectral range of 500–1200 nm. (b) Power-dependent AC photocurrent measured for the 600, 800, and 1000 nm wavelengths, and the corresponding photodetector responsivity, obtained from a typical sample with an illuminated heterostructure area of 13.7 μm^2^. (c) Rise and decay time of the photocurrent.

We now focus on the optoelectronic response of the thick CrSBr/MoS_2_ lateral heterojunction device, which was utilized for the transport measurements. In Figure 4a, we observe a photocurrent at the 10 kHz AC frequency for different illumination wavelengths across a broad spectral range of 500–1200 nm, as a result of the combined light absorption in MoS_2_^5^ and CrSBr.^43^ Over the measured wavelength range, we observe only a positive AC photocurrent, which does not change with the source/drain polarity, further confirming the dominance of the hole conduction in the thick CrSBr/MoS_2_ heterostructure (Supporting Information Section S12). Additionally, we performed power-dependent measurements using 600, 1000, and 800 nm illumination wavelengths, corresponding to the resonant excitations near the absorption edges of MoS_2_, CrSBr, and the midpoint between them, respectively.

Figure 4b illustrates the relationship between the photocurrent (*I*_ph_), responsivity (*R*), and illumination power (*P*). Notably, a nonlinear increase in *R* = *I*_ph_/*P* with a decrease in *P* is a hallmark of photogating, related to the photoconductive gain (calculation of detectivity and gain is in Supporting Information Section S13).^44^ The nonlinearity arises primarily from the carrier lifetime, limited by the recombination rate, and varies with the density of photoexcited carriers. The dominance of the photogating effect is reflected in the power-law relationship, *I*_ph_ ∝ *P*^α^. Typically, α = 1 is associated with a photoconductive effect, while α < 1 indicates photogating.^45^ We observe sublinear behavior in both the low- and high-*P* regimes, where α equals approximately 0.8 and 0.2, respectively. At lower *P*, photogenerated carriers fill up available trapping sites.^2,46^ Once these traps are saturated, additional photogenerated carriers recombine before contributing to the *I*_ph_, indicating a decrease in their average lifetime. Shortening of the lifetime leads to the observed plateau in *R*. With increasing *P*, the built-in electric field becomes screened by the increased carrier density, thereby reducing the efficiency of exciton separation at the junction, which in turn decreases *I*_ph_ generation rate despite the higher photon flux. We achieved a peak value of *R* = 2.4 × 10^5^ A/W using the 800 nm wavelength in the low-power regime.

We also analyzed the rise and decay times of the photodetector device to evaluate its optoelectronic response (Figure 4c). Under AC conditions, the rise (decay) time is 35 ms (70 ms), similar to those typically reported in the DC regime for photogating-dominated 2D materials (Supporting Information Section S14).^2,11,12,20,44,45,47^ In contrast, the time-resolved PL data showed a short exciton decay time of 40 ± 3 ps in the thick CrSBr/MoS_2_ heterostructure, indicating a high PL quenching efficiency through fast radiative processes. The significant discrepancy between the exciton decay time and photocurrent response time can be rationalized by the complex, multistep nature of photocurrent generation. While the time-resolved PL reflects the intrinsic excitonic properties of the heterostructure, photocurrent generation is governed by slower processes, such as charge carrier trapping, transport, and collection at the electrode interface.

## Conclusions

We show that the PL of 1L MoS_2_ can be effectively tuned by interfacing it with CrSBr, whose thickness plays a pivotal role in the tuning process. We observe that CrSBr layers up to ≈20 nm enhance the PL of MoS_2_, while thicker layers result in PL quenching. We rationalize this behavior by a transition from n- to p-type doping in MoS_2_, driven by the charge redistribution at the MoS_2_/CrSBr interface during the thermal equilibration of charge carriers. This phenomenon is further supported by transport measurements, which reveal a correlation between the observed charge-transport properties and the varying thickness of CrSBr flakes. Additionally, we leveraged thick CrSBr/MoS_2_ heterostructure to fabricate a high-performance photodetector, achieving an impressive responsivity of 10^5^ A/W in the AC regime, underscoring its potential for advanced photodetection applications. Our study introduces a simple and controlled method to modulate both the PL and electronic properties of MoS_2_ through interlayer vdW coupling. This approach could be further refined to optimize the PL and electronic properties of MoS_2_ for specific applications. These effects could also be explored for other 2D semiconducting materials and inspire their integration in the next-generation optoelectronic devices, including photodetectors and vertical-cavity surface-emitting lasers, which require unique properties of isotype junctions.

## Materials and Methods

### Sample Preparation

CrSBr bulk crystals were grown by the chemical vapor transport method reported elsewhere.^48^ Flakes of different thicknesses were prepared using the mechanical exfoliation method onto polydimethylsiloxane and subsequently transferred to a clean SiO_2_ (285 nm)/Si substrate. MoS_2_ was exfoliated from commercially available bulk crystals (2D Semiconductors) and transferred on top of the CrSBr flakes and vice versa (for CrSBr/MoS_2_) using a micromechanical transfer stage.

### Microscopy and Spectroscopy

2D crystals were identified using an optical microscope, and their thickness was estimated using the combination of Raman spectroscopy, optical phase contrast, and atomic force microscopy in the PeakForce tapping mode (Bruker Dimension ICON).

KPFM in a frequency-modulation mode was performed with a HORIBA OmegaScope SPM (Horiba Jobin-Yvon) and Au-coated tips (ACCESS-GG-FM, Applied NanoStructures Inc.). The work function of the tip was calibrated using highly ordered pyrolytic graphite.^49^

The Raman and PL spectroscopy measurements were performed on the WITec Alpha 300R instrument in a backscattering geometry. 600 lines/mm (1800 lines/mm) grating was used for the Raman (PL) measurements. The 532 nm excitation laser was focused through a 100× objective (NA = 0.9).

Time-resolved PL measurements were recorded using a custom-built setup coupled to a “PMA Hybrid” Photomultiplier Detector Assembly (Picoquant). A band-pass filter (ET 630/75 nm) was used to record the radiative lifetimes of all the samples. The samples were excited with a 532 nm laser (PDL 800-D, Picoquant) at a frequency of 80 MHz. The decay profiles were analyzed and fitted with a single exponential decay function using the QuCoa software (Picoquant).

### Transport and Optoelectronic Measurements

CrSBr and MoS_2_ flakes were transferred to prepatterned Pt (40 nm)/Ti (10 nm) electrodes, and the measurements were carried out using a semiconductor parameter analyzer (Keithley 2612B or Keithley 4200) under ambient conditions as reported in our previous work.^11,12^ The AC response was measured using an LCR meter (Hioki IM3536) in a two-point (four-wire) probe geometry. All samples subjected to electrical characterization were annealed at 150 °C in 1000 mTorr of Ar atmosphere for 60 min.

All the sample preparation, device fabrication, and measurements were conducted in air at room temperature (295 K), unless stated otherwise.

### Theory

We used density functional theory as implemented in Quantum Espresso to model the band alignment of 1L MoS_2_ and few-layer CrSBr.^50,51^ We used the PBEsol density functional, a plane-wave cutoff energy of 50 Ry, and a charge density cutoff of 500 Ry, with an in-plane Monkhorst–Pack *k*-point grid of 16 × 12 for CrSBr and 8 × 8 for MoS_2_. The lattice parameters of CrSBr were fixed to known experimental values^52^ and the atomic positions fully relaxed until forces were below 0.0001 Ry/bohr; MoS_2_ was fully relaxed to the PBEsol lattice parameter of 3.137 Å. Spin-polarization and Hubbard interaction were taken into account for CrSBr with a Hubbard *U* value of 4 eV, which was empirically chosen to reproduce the experimentally known bond angles^52^ in the monolayer. Spin–orbit coupling was neglected. 2D layers were placed in a 3D periodic box as required by the plane-wave basis set, and the vertical separation was set to a minimum of 20 Å to enable an accurate description of the thin-layer limit and computation of the vacuum level reference energy.

See the Supporting Information Sections S15–S20 for additional Raman and PL spectra of 1L MoS_2_, CrSBr, and 1L MoS_2_/CrSBr heterostructures, fabricated and measured under different conditions along with the transport and DC measurements for another representative device.

## Data Availability

All the data generated and analyzed in this study are included in the Article and its Supporting Information. The data that supports the plots within this paper and other findings of this study are available from the HeyRACK repository at https://doi.org/10.48700/datst.66b2m-xgk50.

## Supporting Information Available

Calculated band structures of 1L MoS_2_ and CrSBr; type II band alignment; KPFM; CrSBr thickness estimation; PL fitting; transport and AC/DC response of the devices; PL and Raman spectra of 1L MoS_2_, CrSBr, and heterostructures prepared under different conditions (PDF)

## References

[ref1] Novoselov K. S.; Geim A. K.; Morozov S. V.; Jiang D.; Zhang Y.; Dubonos S. V.; Grigorieva I. V.; Firsov A. A. Electric Field Effect in Atomically Thin Carbon Films. Science 2004, 306, 666–669. 10.1126/science.1102896. 15499015

[ref2] Lopez-Sanchez O.; Lembke D.; Kayci M.; Radenovic A.; Kis A. Ultrasensitive Photodetectors Based on Monolayer MoS_2_. Nat. Nanotechnol. 2013, 8, 497–501. 10.1038/nnano.2013.100. 23748194

[ref3] Korn T.; Heydrich S.; Hirmer M.; Schmutzler J.; Schüller C. Low-temperature Photocarrier Dynamics in Monolayer MoS_2_. Appl. Phys. Lett. 2011, 99, 102109 10.1063/1.3636402.

[ref4] Wang Q. H.; Kalantar-Zadeh K.; Kis A.; Coleman J. N.; Strano M. S. Electronics and Optoelectronics of Two-Dimensional Transition Metal Dichalcogenides. Nat. Nanotechnol. 2012, 7, 699–712. 10.1038/nnano.2012.193. 23132225

[ref5] Splendiani A.; Sun L.; Zhang Y.; Li T.; Kim J.; Chim C.; Galli G.; Wang F. Emerging Photoluminescence in Monolayer MoS_2_. Nano Lett. 2010, 10, 1271–1275. 10.1021/nl903868w. 20229981

[ref6] Mak K. F.; Lee C.; Hone J.; Shan J.; Heinz T. F. Atomically Thin MoS_2_: A New Direct-gap Semiconductor. Phys. Rev. Lett. 2010, 105, 136805 10.1103/PhysRevLett.105.136805. 21230799

[ref7] Mak K. F.; He K.; Lee C.; Lee G. H.; Hone J.; Heinz T. F.; Shan J. Tightly Bound Trions in Monolayer MoS_2_. Nat. Mater. 2013, 12, 207–211. 10.1038/nmat3505. 23202371

[ref8] Jones A. M.; Yu H.; Ghimire N. J.; Wu S.; Aivazian G.; Ross J. S.; Zhao B.; Yan J.; Mandrus D. G.; Xiao D.; et al. Optical Generation of Excitonic Valley Coherence in Monolayer WSe_2_. Nat. Nanotechnol. 2013, 8, 634–638. 10.1038/nnano.2013.151. 23934096

[ref9] Ross J. S.; Wu S.; Yu H.; Ghimire N. J.; Jones A. M.; Aivazian G.; Yan J.; Mandrus D. G.; Xiao D.; Yao W.; et al. Electrical Control of Neutral and Charged Excitons in a Monolayer Semiconductor. Nat. Commun. 2013, 4, 1474 10.1038/ncomms2498. 23403575

[ref10] Mak K. F.; He K.; Shan J.; Heinz T. F. Control of Valley Polarization in Monolayer MoS_2_ by Optical Helicity. Nat. Nanotechnol. 2012, 7, 494–498. 10.1038/nnano.2012.96. 22706698

[ref11] Sahu S.; Panda J.; Haider G.; Frank O.; Kalbáč M.; Velický M. Self-biased High-responsivity Photodetector Based on a Bi_2_SeTe_2_ Topological Insulator. ACS Appl. Electron. Mater. 2023, 5, 6697–6703. 10.1021/acsaelm.3c01195.

[ref12] Panda J.; Sahu S.; Haider G.; Thakur M. K.; Mosina K.; Velický M.; Vejpravova J.; Sofer Z.; Kalbáč M. Polarization-resolved Position-sensitive Self-powered Binary Photodetection in Multilayer Janus CrSBr. ACS Appl. Mater. Interfaces 2024, 16, 1033–1043. 10.1021/acsami.3c13552. 38147583 PMC10788859

[ref13] Novoselov K. S.; Mishchenko A.; Carvalho A.; Castro Neto A. 2D Materials and van der Waals Heterostructures. Science 2016, 353, aac9439 10.1126/science.aac9439. 27471306

[ref14] Xia F.; Wang H.; Xiao D.; Dubey M.; Ramasubramaniam A. Two-dimensional Material Nanophotonics. Nat. Photonics 2014, 8, 899–907. 10.1038/nphoton.2014.271.

[ref15] Duan J.; Chava P.; Ghorbani-Asl M.; Erb D.; Hu L.; Krasheninnikov A. V.; Schneider H.; Rebohle L.; Erbe A.; Helm M.; et al. Enhanced Trion Emission in Monolayer MoSe_2_ by Constructing a Type-I van der Waals Heterostructure. Adv. Funct. Mater. 2021, 31, 2104960 10.1002/adfm.202104960.

[ref16] Ramos M.; Marques-Moros F.; Esteras D. L.; Mañas-Valero S.; Henríquez-Guerra E.; Gadea M.; Baldoví J. J.; Canet-Ferrer J.; Coronado E.; Calvo M. R. Photoluminescence Enhancement by Band Alignment Engineering in MoS_2_/FePS_3_ Van der Waals Heterostructures. ACS Appl. Mater. Interfaces 2022, 14, 33482–33490. 10.1021/acsami.2c05464. 35839147 PMC9335528

[ref17] Serati de Brito C.; Faria Junior P. E.; Ghiasi T. S.; Ingla-Aynés J.; Rabahi C. R.; Cavalini C.; Dirnberger F.; Mañas-Valero S.; Watanabe K.; Taniguchi T.; et al. Charge Transfer and Asymmetric Coupling of MoSe_2_ Valleys to the Magnetic Order of CrSBr. Nano Lett. 2023, 23, 11073–11081. 10.1021/acs.nanolett.3c03431. 38019289

[ref18] Bellus M. Z.; Li M.; Lane S. D.; Ceballos F.; Cui Q.; Zeng X. C.; Zhao H. Type-I Van der Waals Heterostructure Formed by MoS_2_ and ReS_2_ Monolayers. Nanoscale Horizons 2017, 2, 31–36. 10.1039/C6NH00144K. 32260674

[ref19] Xiao J.; Zhang L.; Zhou H.; Shao Z.; Liu J.; Zhao Y.; Li Y.; Liu X.; Xie H.; Gao Y.; et al. Type-II Interface Band Alignment in the vdW PbI_2_–MoSe_2_ Heterostructure. ACS Appl. Mater. Interfaces 2020, 12, 32099–32105. 10.1021/acsami.0c04985. 32603081

[ref20] Sahu S.; Haider G.; Rodriguez A.; Plšek J.; Mergl M.; Kalbáč M.; Frank O.; Velický M. Large-area Mechanically-exfoliated Two-dimensional Materials on Arbitrary Substrates. Adv. Mater. Technol. 2023, 8, 2201993 10.1002/admt.202201993.

[ref21] Wilson N. P.; Lee K.; Cenker J.; Xie K.; Dismukes A. H.; Telford E. J.; Fonseca J.; Sivakumar S.; Dean C.; Cao T.; et al. Interlayer Electronic Coupling on Demand in a 2D Magnetic Semiconductor. Nat. Mater. 2021, 20, 1657–1662. 10.1038/s41563-021-01070-8. 34312534

[ref22] Wang T.; Zhang D.; Yang S.; Lin Z.; Chen Q.; Yang J.; Gong Q.; Chen Z.; Ye Y.; Liu W. Magnetically-dressed CrSBr Exciton-polaritons in Ultrastrong Coupling Regime. Nat. Commun. 2023, 14, 59066 10.1038/s41467-023-41688-7. PMC10520032 37749106

[ref23] Mouri S.; Miyauchi Y.; Matsuda K. Tunable Photoluminescence of Monolayer MoS_2_ via Chemical Doping. Nano Lett. 2013, 13, 5944–5948. 10.1021/nl403036h. 24215567

[ref24] Park Y.; Li N.; Jung D.; Singh L. T.; Baik J.; Lee E.; Oh D.; Kim Y. D.; Lee J. Y.; Woo J.; et al. Unveiling the Origin of n-type Doping of Natural MoS_2_: Carbon. npj 2D Mater. Appl. 2023, 7, 60 10.1038/s41699-023-00424-x.

[ref25] Vaquero D.; Clericò V.; Salvador-Sánchez J.; Martín-Ramos A.; Díaz E.; Domínguez-Adame F.; Meziani Y. M.; Diez E.; Quereda J. Excitons Trions and Rydberg States in Monolayer MoS_2_ Revealed by Low-Temperature Photocurrent Spectroscopy. Commun. Phys. 2020, 3, 194 10.1038/s42005-020-00460-9.

[ref26] Godde T.; Schmidt D.; Schmutzler J.; Aßmann M.; Debus J.; Withers F.; Alexeev E.; Del Pozo-Zamudio O.; Skrypka O.; Novoselov K.; et al. Exciton and Trion Dynamics in Atomically Thin MoSe_2_ and WSe_2_: Effect of Localization. Phys. Rev. B 2016, 94, 165301 10.1103/physrevb.94.165301.

[ref27] Singh A.; Moody G.; Tran K.; Scott M. E.; Overbeck V.; Berghäuser G.; Schaibley J.; Seifert E. J.; Pleskot D.; Gabor N. M.; et al. Trion Formation Dynamics in Monolayer Transition Metal Dichalcogenides. Phys. Rev. B 2016, 93, 041401 10.1103/physrevb.93.041401.

[ref28] Lee Y.; Ghimire G.; Roy S.; Kim Y.; Seo C.; Sood A.; Jang J. I.; Kim J. Impeding Exciton–exciton Annihilation in Monolayer WS_2_ by Laser Irradiation. ACS Photonics 2018, 5, 2904–2911. 10.1021/acsphotonics.8b00249.

[ref29] Lien D. H.; Uddin S. Z.; Yeh M.; Amani M.; Kim H.; Ager III J. W.; Yablonovitch E.; Javey A. Electrical Suppression of All Nonradiative Recombination Pathways in Monolayer Semiconductors. Science 2019, 364, 468–471. 10.1126/science.aaw8053. 31048488

[ref30] Yu Y.; Lee D.; Jeong B. The Dependence of the Work Function of Pt (111) on Surface Carbon Investigated with near Ambient Pressure X-ray Photoelectron Spectroscopy. Appl. Surf. Sci. 2023, 607, 155005 10.1016/j.apsusc.2022.155005.

[ref31] Kim J. H.; Lee J.; Kim J. H.; Hwang C.; Lee C.; Park J. Y. Work Function Variation of MoS_2_ Atomic Layers Grown with Chemical Vapor Deposition: The Effects of Thickness and the Adsorption of Water/Oxygen Molecules. Appl. Phys. Lett. 2015, 106, 251606 10.1063/1.4923202.

[ref32] Smolenski S.; Wen M.; Li Q.; Downey E.; Alfrey A.; Liu W.; Kondusamy A. L. N.; Bostwick A.; Jozwiak C.; Rotenberg E.; Zhao L.; Deng H.; Lv B.; Zgid D.; Gull E.; Jo N. H. Large Exciton Binding Energy in a Bulk van der Waals Magnet from Quasi-1D Electronic Localization. Nat. Commun. 2025, 16, 1134 10.1038/s41467-025-56457-x. 39880826 PMC11779854

[ref33] Guo Y.; Li J.; Zhan X.; Wang C.; Li M.; Zhang B.; Wang Z.; Liu Y.; Yang K.; Wang H.; et al. Van der Waals Polarity-engineered 3D Integration of 2D Complementary Logic. Nature 2024, 630, 346–352. 10.1038/s41586-024-07438-5. 38811731 PMC11168927

[ref34] Klein J.; Pingault B.; Florian M.; Heißenbüttel M. C.; Steinhoff A.; Song Z.; Torres K.; Dirnberger F.; Curtis J. B.; Weile M.; et al. The Bulk van der Waals Layered Magnet CrSBr is a Quasi-1D Material. ACS Nano 2023, 17, 5316–5328. 10.1021/acsnano.2c07316. 36926838

[ref35] Nourbakhsh A.; Zubair A.; Dresselhaus M. S.; Palacios T. Transport Properties of a MoS_2_/WSe_2_ Heterojunction Transistor and its Potential for Application. Nano Lett. 2016, 16, 1359–1366. 10.1021/acs.nanolett.5b04791. 26784325

[ref36] Wei L.; Wu Z.; Wei Y.; Li C.; Fu Z.; Han J.; Yang X.; Xie J.; Tian Z.; Zhou H.; et al. High-gain and Tunable Linear Photodetection in 2D Tunneling Heterostructures Through Potential Engineering. Adv. Funct. Mater. 2024, 34, 2411736 10.1002/adfm.202411736.

[ref37] Klein J.; Song Z.; Pingault B.; Dirnberger F.; Chi H.; Curtis J. B.; Dana R.; Bushati R.; Quan J.; Dekanovsky L.; et al. Sensing the Local Magnetic Environment Through Optically Active Defects in a Layered Magnetic Semiconductor. ACS Nano 2022, 17, 288–299. 10.1021/acsnano.2c07655. 36537371

[ref38] Heine V. Theory of Surface States. Phys. Rev. 1965, 138, A1689 10.1103/PhysRev.138.A1689.

[ref39] Monch W. On the Physics of Metal-semiconductor Interfaces. Rep. Prog. Phys. 1990, 53, 221 10.1088/0034-4885/53/3/001.

[ref40] Kobayashi N.; Masumoto H.; Takahashi S.; Maekawa S. Giant Dielectric and Magnetoelectric Responses in Insulating Nanogranular Films at Room Temperature. Nat. Commun. 2014, 5, 4417 10.1038/ncomms5417. 25048805 PMC4109019

[ref41] Biswas R.; Sinha C. Quenching Effect of Oscillating Potential on Anisotropic Resonant Transmission Through a Phosphorene Electrostatic Barrier. Sci. Rep. 2021, 11, 2881 10.1038/s41598-021-82323-z. 33536502 PMC7859226

[ref42] Boev M.; Kovalev V.; Kibis O. Optically Induced Resonant Tunneling of Electrons in Nanostructures. Sci. Rep. 2023, 13, 19625 10.1038/s41598-023-46998-w. 37949951 PMC10638321

[ref43] Linhart W.; Rybak M.; Birowska M.; Scharoch P.; Mosina K.; Mazanek V.; Kaczorowski D.; Sofer Z.; Kudrawiec R. Optical Markers of Magnetic Phase Transition in CrSBr. J. Mater. Chem. C 2023, 11, 8423–8430. 10.1039/D3TC01216F.

[ref44] Konstantatos G.; Badioli M.; Gaudreau L.; Osmond J.; Bernechea M.; De Arquer F. P. G.; Gatti F.; Koppens F. H. Hybrid Graphene–quantum Dot Phototransistors with Ultrahigh Gain. Nat. Nanotechnol. 2012, 7, 363–368. 10.1038/nnano.2012.60. 22562036

[ref45] Island J. O.; Blanter S. I.; Buscema M.; van der Zant H. S.; Castellanos-Gomez A. Gate Controlled Photocurrent Generation Mechanisms in High-gain In_2_Se_3_ Phototransistors. Nano Lett. 2015, 15, 7853–7858. 10.1021/acs.nanolett.5b02523. 26540135

[ref46] Konstantatos G.; Clifford J.; Levina L.; Sargent E. H. Sensitive Solution-processed Visible-wavelength Photodetectors. Nat. Photonics 2007, 1, 531–534. 10.1038/nphoton.2007.147.

[ref47] Khan S.; Khan A.; Azadmanjiri J.; Kumar Roy P.; Děkanovský L.; Sofer Z.; Numan A. 2D Heterostructures for Highly Efficient Photodetectors: From Advanced Synthesis to Characterizations, Mechanisms, and Device Applications. Adv. Photonics Res. 2022, 3, 2100342 10.1002/adpr.202100342.

[ref48] Klein J.; Pham T.; Thomsen J.; Curtis J.; Denneulin T.; Lorke M.; Florian M.; Steinhoff A.; Wiscons R.; Luxa J.; et al. Control of Structure and Spin Texture in the Van der Waals Layered Magnet CrSBr. Nat. Commun. 2022, 13, 5420 10.1038/s41467-022-32737-8. 36109520 PMC9478124

[ref49] Fernández Garrillo P. A.; Grévin B.; Chevalier N.; Borowik Ł. Calibrated Work Function Mapping by Kelvin Probe Force Microscopy. Rev. Sci. Instrum. 2018, 89, 043702 10.1063/1.5007619. 29716375

[ref50] Giannozzi P.; Baroni S.; Bonini N.; Calandra M.; Car R.; Cavazzoni C.; Ceresoli D.; Chiarotti G. L.; Cococcioni M.; Dabo I.; Dal Corso A.; de Gironcoli S.; Fabris S.; Fratesi G.; Gebauer R.; Gerstmann U.; Gougoussis C.; Kokalj A.; Lazzeri M.; Martin-Samos L.; et al. QUANTUM ESPRESSO: A Modular and Open-source Software Project for Quantum Simulations of Materials. J. Phys.: Condens. Matter 2009, 21 (19pp), 395502 10.1088/0953-8984/21/39/395502. 21832390

[ref51] Giannozzi P.; Andreussi O.; Brumme T.; Bunau O.; Buongiorno Nardelli M.; Calandra M.; Car R.; Cavazzoni C.; Ceresoli D.; Cococcioni M.; Colonna N.; Carnimeo I.; Dal Corso A.; de Gironcoli S.; Delugas P.; DiStasio R. A. Jr.; Ferretti A.; Floris A.; Fratesi G.; Fugallo G.; et al. Advanced Capabilities for Materials Modelling with QUANTUM ESPRESSO. J. Phys.: Condens. Matter 2017, 29, 465901 10.1088/1361-648x/aa8f79. 29064822

[ref52] Boix-Constant C.; Mañas-Valero S.; Ruiz A. M.; Rybakov A.; Konieczny K. A.; Pillet S.; Baldoví J. J.; Coronado E. Probing the Spin Dimensionality in Single-layer CrSBr Van der Waals Heterostructures by Magneto-transport Measurements. Adv. Mater. 2022, 34, 2204940 10.1002/adma.202204940. 36008364

