# Supplementary material for: Tuning of MoS2 Photoluminescence in Heterostructures with CrSBr
Source: ACS Appl Mater Interfaces. 2025 Apr 15;17(17):25693–701. doi: 10.1021/acsami.5c01924 (PMC12051177; doi:10.1021/acsami.5c01924)
Supplement: Supplementary file 1 — am5c01924_si_001.pdf [file am5c01924_si_001.pdf]

Supporting Information: Tuning of MoS_2 Photoluminescence in Heterostructures with CrSBr

Satyam Sahu,[†,‡,@] Oleksandr Volochanskyi,[†,¶,@] Vaibhav Varade,[§] Luka Pirker,[†] Viktor Zólyomi,[‖] János Koltai,[⊥] Kseniia Mosina,[#] Zdeněk Sofer,[#] Otakar Frank,[*,†] Jana Vejpravová,[§] Martin Kalbáč,[†] and Matěj Velický[*,†]

†*J. Heyrovský Institute of Physical Chemistry, Czech Academy of Sciences, Dolejškova 2155/3, 182 23 Prague, Czech Republic*

‡*Department of Biophysics, Chemical and Macromolecular Physics, Faculty of Mathematics and Physics, Charles University, Ke Karlovu 3, 121 16 Prague, Czech Republic*

¶*Department of Physical Chemistry, Faculty of Chemical Engineering, University of Chemistry and Technology in Prague, Technická 5, 142 00 Prague, Czech Republic*

§*Department of Condensed Matter Physics, Faculty of Mathematics and Physics, Charles University, Ke Karlovu 5, 121 16 Prague, Czech Republic*

‖*Hartree Centre, STFC Daresbury Laboratory, Daresbury WA4 4AD, United Kingdom*

⊥*Department of Biological Physics, Eötvös Loránd University, Pázmány Péter sétány 1/A, Budapest 1117, Hungary*

#*Department of Inorganic Chemistry, University of Chemistry and Technology Prague, Technická 5, 166 28 Prague, Czech Republic*

@*Contributed equally to this work*

E-mail: otakar.frank@jh-inst.cas.cz; matej.velicky@jh-inst.cas.cz

Table of contents

S1. Density Functional Theory and Band Alignment S-3
S2. Type II Band Alignment from Experimental Data S-5
S3. Kelvin Probe Force Microscopy S-6
S4. CrSBr Thickness Estimation S-7
S5. Photoluminescence Fitting Procedure and Fit Parameters S-7
S6. Transport Measurements S-9
S7. DC Response of the Device S-10
S8. Transfer Characteristics of the Lateral Junction Device S-11
S9. Frequency- and Amplitude-Dependent AC Response of the Device S-13
S10. Amplitude-Dependent Capacitance Change S-14
S11. Summary of AC Characteristics of the Device S-15
S12. Photocurrent Independence of the Source/Drain Polarity S-18
S13. Detectivity and Gain S-19
S14. Figure of Merit Comparison with the State-of-the-Art Systems S-20
S15. Photoluminescence of CrSBr S-21
S16. Raman Spectra of CrSBr S-21
S17. CrSBr Thickness-Dependent Raman Spectra of MoS_2 . . . . S-22
S18. Interference Effect S-24
S19. Environmental Effect S-25
S20. Current-Voltage and Transfer Characteristics of another Vertical Junction Device . S-26

S1. Density Functional Theory and Band Alignment

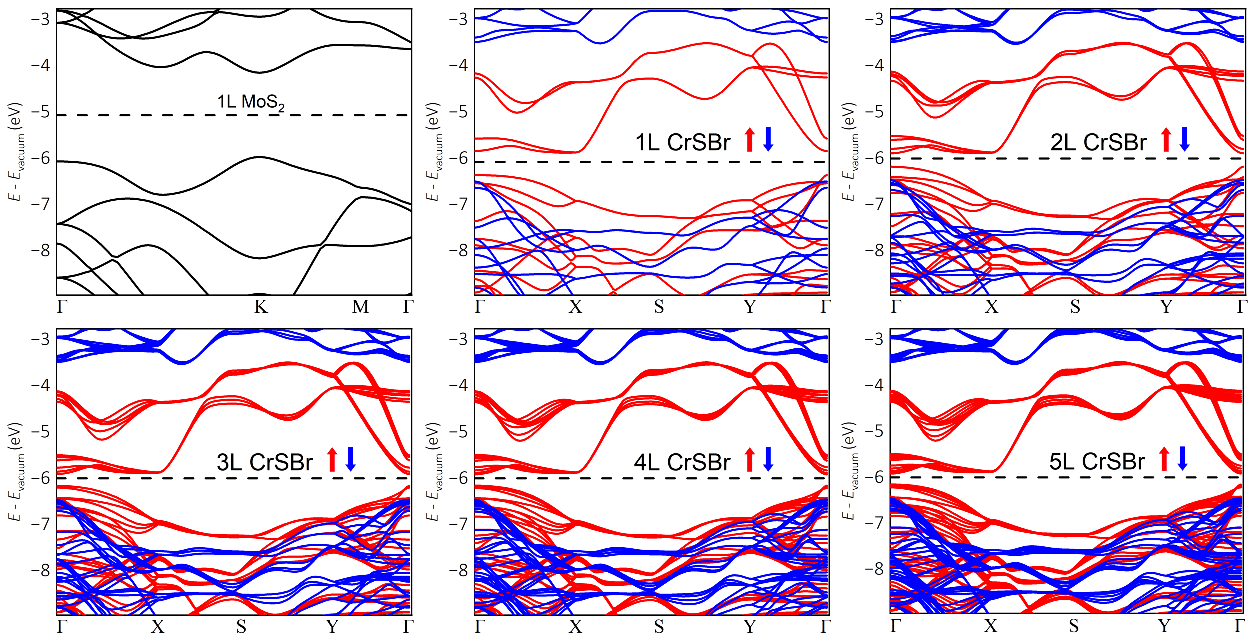

Figure S1: Calculated band structures for 1L MoS_2 and 1L-5L CrSBr as indicated in the individual panels. The red and blue curves show the spin-up and spin-down bands, respectively.

To gain insights into the plausible band alignment between MoS_2 and bulk CrSBr, we investigated the band edge positions and the band gaps using first-principles density functional theory (DFT) calculations for freestanding 1L MoS_2 and 1L–5L CrSBr. The calculated band structures for both materials are shown in Figure S1. One can see that 1L MoS_2 has a direct band gap of around 1.8 eV at the K point in the Brillouin zone. The direct band gap in CrSBr is at the Γ point, in line with previous reports.[1] Next, we extracted the positions of the conduction band minimum (CBM), Fermi level (E_F), and valence band maximum (VBM) with respect to the vacuum level for CrSBr, and plotted them as a function of the number of layers (Figure S2). Our calculations indicate that 5L is a good approximation for bulk CrSBr since the energy levels nearly converge. Furthermore, in Figure S3, we plot the band positions of 1L MoS_2 with respect to 5L-CrSBr and confirm the type II band alignment. Note that the calculations underestimate the band gaps of both MoS_2 and CrSBr, as expected for a semilocal DFT when compared to the GW-approximation-based calculations or experimental measurements (1.9-2.8 eV for MoS_2[2] and 1.5-2.1 eV for CrSBr[3]), meaning that the

exact position of the CBM with respect to the vacuum level is subject to uncertainty. However, the alignment of the ground state VBM energies is expected to be accurate.

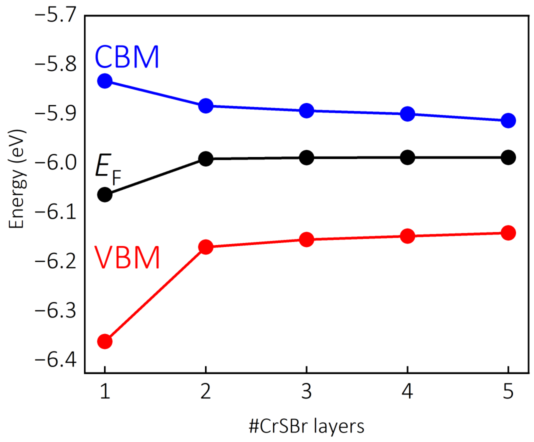

Figure S2: Calculated CBM, E_F, and VBM values with respect to the vacuum level as a function of the CrSBr thickness.

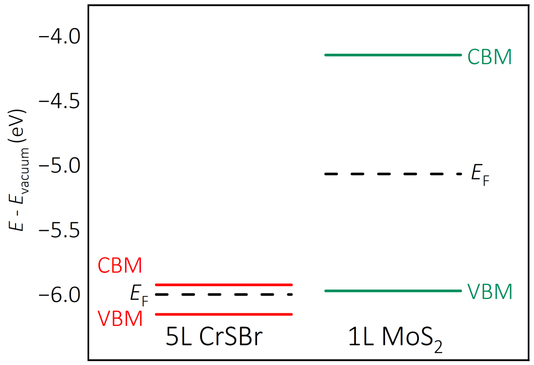

Figure S3: Type-II band alignment for 5L-CrSBr and 1L MoS_2 predicted by DFT.

S2. Type II Band Alignment from Experimental Data

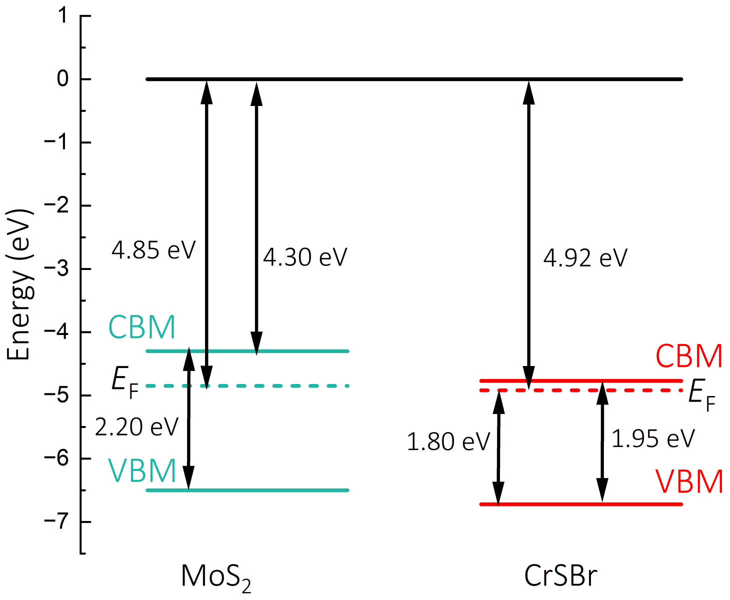

Figure S4: Type-II band alignment from experimental data.

We also estimated the band alignment using the experimental data available for 1L MoS_2 and CrSBr. We utilized the reported electron affinity (∼4.3 eV) and electronic band gap (∼2.2 eV)[4–6] to estimate the VBM and CBM positions in 1L MoS_2, and considered the work function of 4.85 eV from our KPFM measurements to determine the E_F. For CrSBr, we used the VBM position from μ-ARPES measurements, which was reported to be 1.8 eV below E_F[7] and the measured electronic band gap of ∼ 1.95 eV[7,8] along with the work function of 4.92 eV. Figure S4 summarizes the experimental data and confirms the type II band alignment for 1L-MoS_2/CrSBr heterostructures.

S3. Kelvin Probe Force Microscopy

Kelvin probe force microscopy measurements were performed using an Au-coated tip. The measurements were calibrated using the surface of a freshly cleaved highly oriented pyrolytic graphite (HOPG), and the work function of the tip (ϕ_{tip}) was calculated from the measured contact potential difference and the reported work function of HOPG (ϕ_{HOPG} = 4.6 eV):[9]

$$\phi_{\text{tip}} = \phi_{\text{HOPG}} + e \cdot \Delta V_{\text{cpd}} = 4.6 \text{ eV} + e \cdot 0.3 \text{ V} = 4.9 \text{ eV}$$

where ΔV_{cpd} is the contact potential difference and e is the elementary charge.

Thus, the work functions of MoS_2 on SiO_2/Si and MoS_2 in proximity to CrSBr (ϕ_{MoS_2}) were obtained as:

$$\phi_{MoS_2} = \phi_{\text{tip}} - \Delta V_{\text{cpd}}$$

Figure S5 shows the work function map of bare 1L MoS_2. Note that the work function of MoS_2 can vary significantly due to the crystalline disorder, interactions with the substrate, and adventitious contamination.[10–12]

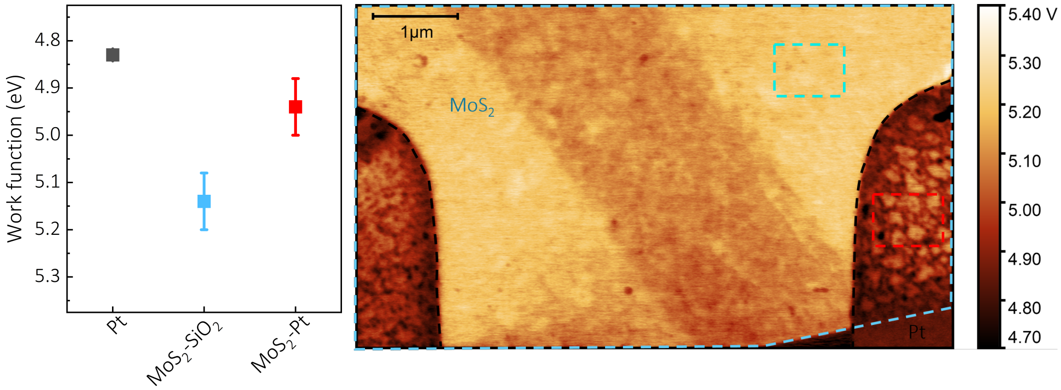

Figure S5: Work function spatial map of bare 1L MoS_2.

S4. CrSBr Thickness Estimation

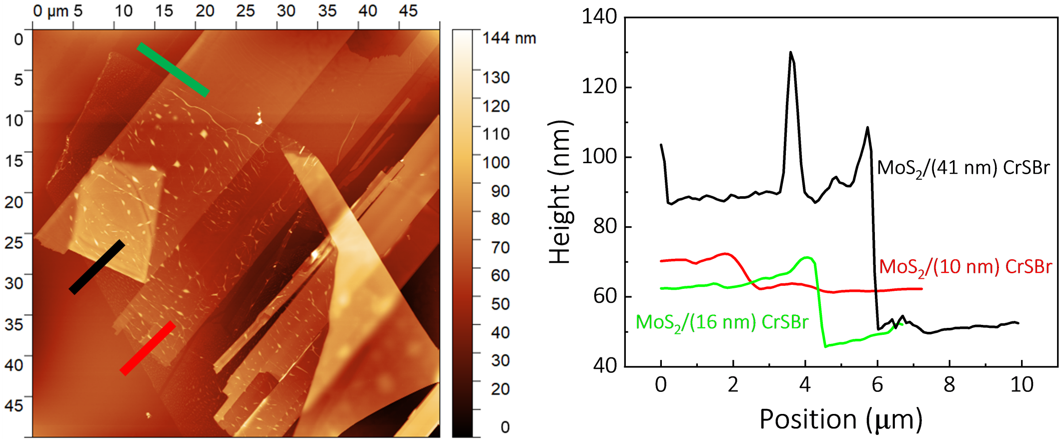

Figure S6: Atomic force microscopy topographic image (left) of the representative sample showed in the main text and the corresponding height profiles (right).

In Figure S6 (left panel), we show the topography of the sample measured by atomic force microscopy. Colored solid curves correspond to the CrSBr thickness profiles shown in the right panel of Figure S6. The spikes arise from the nanobubbles formed during the heterostructure fabrication.[13]

S5. Photoluminescence Fitting Procedure and Fit Parameters

Figure S7 details the fitting of the photoluminescence (PL) spectra in different samples. Since the emission is broadened as a result of the thermal fluctuations at room temperature, we used the Voigt function to fit the data, instead of the Lorentzian.

We fit the bare 1L MoS_2 spectra with three peaks corresponding to the A^- trion, A^0, and B^0 excitons, respectively. We do not fit the B^0 exciton in the heterostructure regions, since it is not clearly resolved. Table S1 shows the fit parameters for the sample shown in the main text. As expected, there is a separation of around 30–40 meV between the trionic and excitonic components, which is in line with the room temperature trion binding energy reported in the literature.[14] The variations in the spectral weights indicate the possible role of the charge transfer from MoS_2 to CrSBr.

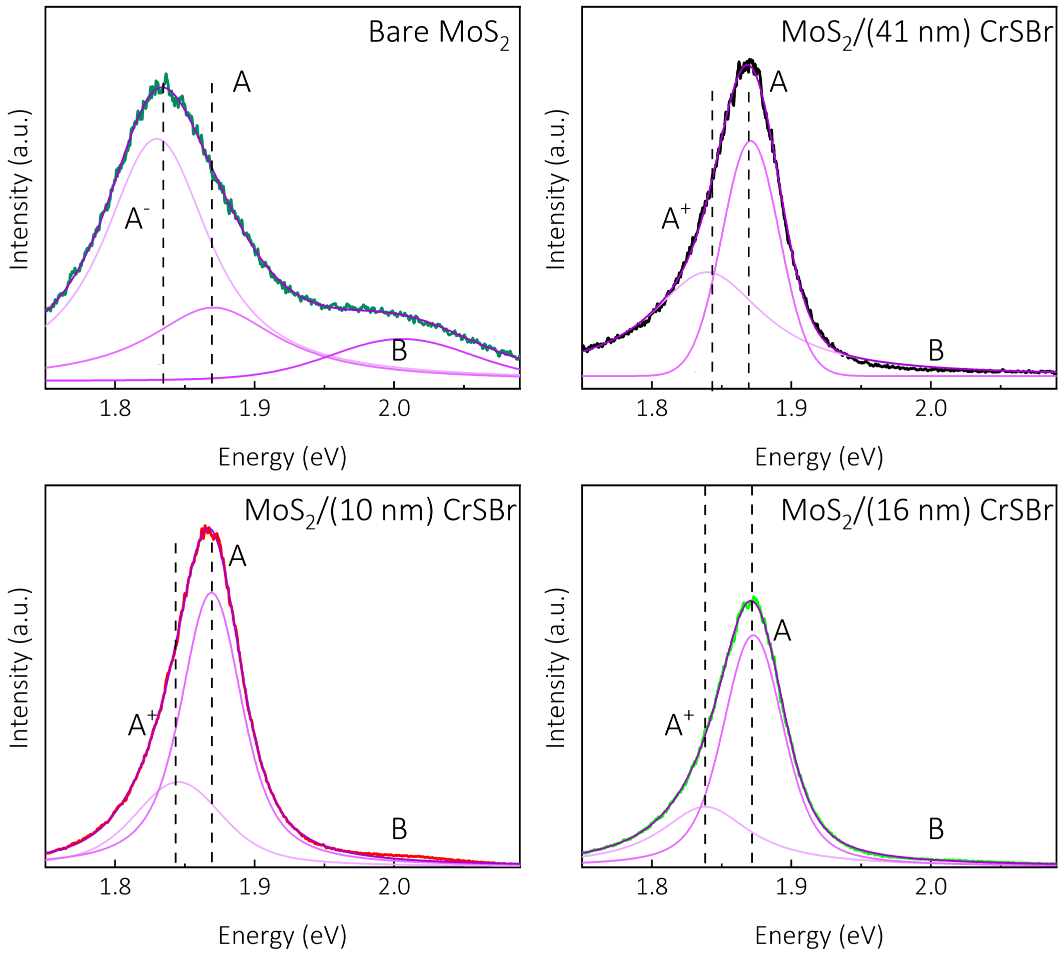

Figure S7: PL spectra and fits using the Voigt function for the samples presented in the manuscript, as marked in the individual panels. Pink curves are the deconvoluted $A^{-/+}$, A^0, and B^0 components.

Table S1: Fitted values of the peak positions and integrated area for the $A^{-/+}$ and A^0 components of the MoS_2 spectra shown in Figure S7.

	Position (eV)		Area (a.u.)	
	$A^{-/+}$	A^0	$A^{-/+}$	A^0
Bare MoS_2	1.83	1.87	138	55
MoS_2/(10 nm) CrSBr	1.84	1.87	195	460
MoS_2/(16 nm) CrSBr	1.84	1.87	116	238
MoS_2/(41 nm) CrSBr	1.84	1.87	49	36

S6. Transport Measurements

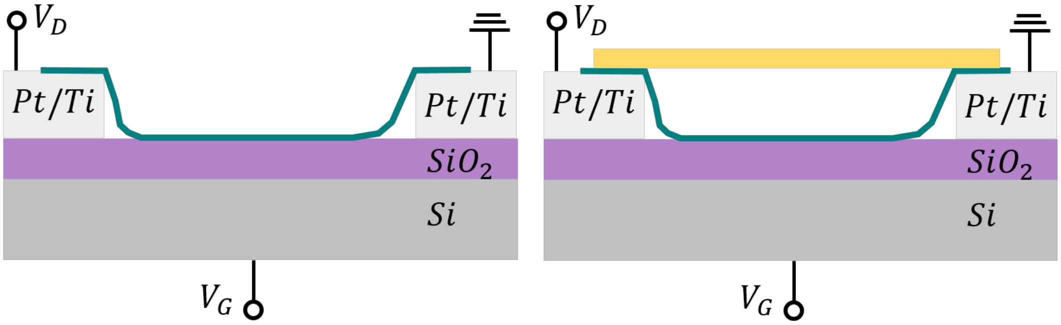

Figure S8: Device schematic for transport measurements. Left panel: 1L MoS_2 (teal) on SiO_2. Right panel: 1L MoS_2 in contact with CrSBr (yellow).

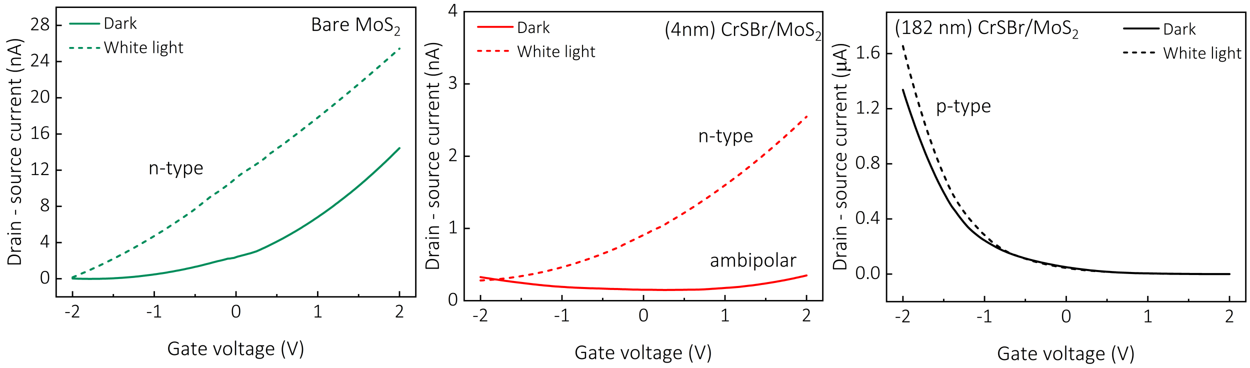

Figure S9: Transfer characteristics of bare MoS_2 and CrSBr/MoS_2 devices under dark and illumination conditions at drain-source voltage $V_{ds} = 2$ V.

We performed transport experiments in the back-gated geometry to verify the majority of carriers in the individual systems. Two samples were prepared, representing bare MoS_2 on SiO_2 and CrSBr on top of MoS_2. In the latter case, only MoS_2 was in contact with the source and drain electrodes while CrSBr laid on top of bare MoS_2, as shown in Figure S8 (right panel).

For all the samples, measurements were conducted in the dark and under white light illumination. For bare MoS_2, we clearly see the typical n-type behavior, shown in Figure S9 (left panel), for both conditions. For MoS_2 in contact with a thin CrSBr flake, initially in the dark, the behavior was ambipolar, suggesting that the excess of electrons in MoS_2 was partially depleted to thin CrSBr (middle panel). However, under illumination, the system reverts to dominant n-type transport.

This shift suggests that photogenerated electrons are more mobile and effective in MoS_2, leading to enhanced electron conduction. The thin CrSBr likely acts as a weak perturbation, allowing MoS_2's natural n-type behavior to dominate under light exposure due to more efficient photocarrier generation in the conduction band of MoS_2. Next, for the thick CrSBr flake on top of MoS_2, the majority of the electrons move to CrSBr, thanks to the larger number of available vacant states, making MoS_2 strongly p-doped by the presence of excess holes (right panel).

S7. DC Response of the Device

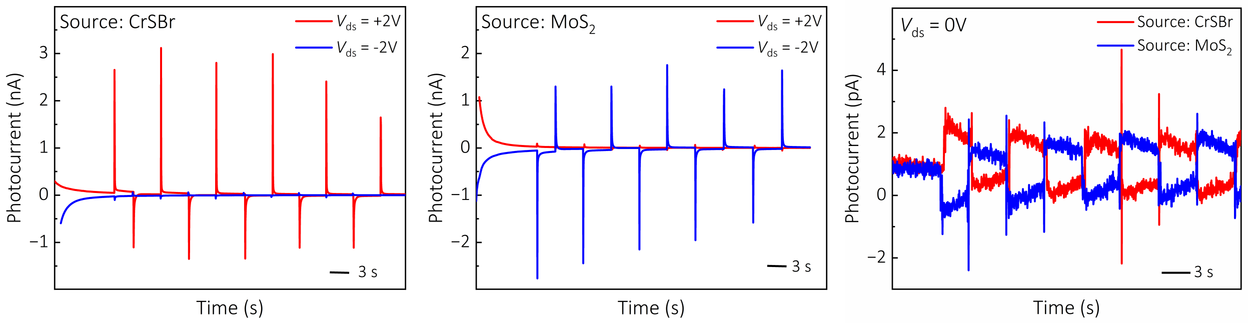

Figure S10: Summary of the time-dependent DC photoresponse of thick CrSBr/MoS_2 lateral junction device under different external bias conditions. Biased response using CrSBr as a source (left panel) and MoS_2 as a source (middle panel). Photoresponse under 0 V bias (right panel).

During the initial phase of illumination under DC conditions, the photocurrent in the external circuit increases progressively as the photogenerated carriers are separated and transported. An internal electric field is established when holes are captured at the interface, accelerating photogenerated electrons toward recombination with these trapped holes. This is reflected as a rapid decay in the photocurrent from its peak down to a steady-state level, determined by the external voltage (Figure S10). Thus, the initial surge in the photocurrent is attributed to the charging of the space charge capacitance region or displacement current caused by the movement of photogenerated holes toward the heterojunction.[15]

When the light source is turned off, the build-up of holes at the heterojunction stops almost instantaneously, but the holes trapped at the interface continue to recombine with free electrons. This delayed recombination process induces a current opposite to that observed during illumination.

These processes lead to a characteristic overshoot in the transient photocurrent.[16]

A similar behavior is observed at zero bias, albeit without the rapid transient response due to a lack of a significant external voltage to drive the carrier separation. The absence of external voltage limits the driving force for the fast carrier diffusion, leading to a more gradual photoresponse.

These measurements also reveal that the thick CrSBr/MoS_2 device retains similar rectifying properties as the thin sample (described in the main text). However, the charging feedback masks this effect, as evident in I_{ds}–V_{ds} curves.

S8. Transfer Characteristics of the Lateral Junction Device

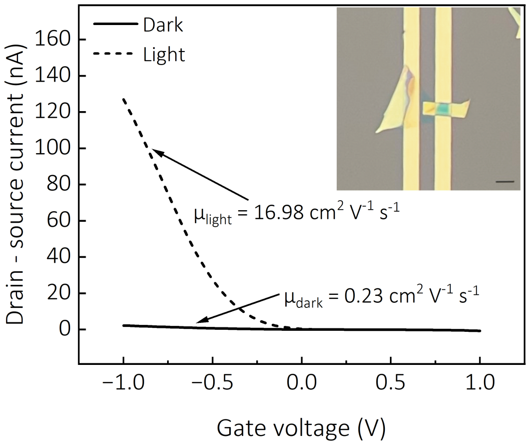

Figure S11: Transfer characteristics of a thick CrSBr/MoS_2 device under dark and illumination at V_{ds} = 2 V. Inset shows an optical image of the device. The scale bar corresponds to 5 μm.

To confirm the majority of charge carriers in the lateral junction device, we conduct additional transfer characteristic measurements on the same sample used to describe the AC-driven photodetector in the main text. The pronounced p-type conduction observed in the transport measurements (Figure S11) highlights the dominant role of holes in the charge transport across the thick CrSBr/MoS_2 lateral junction. In this device, where MoS_2 (CrSBr) serves as the drain (source), the net carrier mobility of holes under illumination is significantly higher than that of electrons. The I_{ds} gradually decreases as the gate voltage changes from negative to positive, proving that holes are the majority carriers in the heterostructure. We extract the charge carrier mobility (μ) from the

following relation:

$$\mu = \frac{L}{W} \times \frac{dI_{\text{ds}}}{dV_{\text{g}}} \times \frac{1}{C_{\text{bg}} V_{\text{ds}}}$$

where *L* and *W* are the length and the width of the active heterostructure area, respectively, C_{bg} is the back gate capacitance (11.6 nF/cm^2), V_{ds} is the bias voltage, and $dI_{\text{ds}}/dV_{\text{g}}$ is the transconductance extracted using the transconductance change method.[17] Upon white light illumination, the hole mobility (μ^+) increases dramatically by a factor of 74, while the electron mobility (μ^-) is slightly reduced. We also calculated the density of holes (n^+) and electrons (n^-) using the following equation:

$$n^{+/-} = \frac{C_{\text{bg}}(V_{\text{g}} - V_{\text{th}})}{e}$$

where V_{th} is the threshold voltage determined by the transconductance change method (–0.4 V in the dark and –0.5 V under illumination). The calculated values are summarized in Table S2.

Table S2: Summary of the extracted charge carrier mobilities and densities in the thick CrSBr/MoS_2 device in the dark and under illumination.

	μ^+ (cm^2 V^{-1} s^{-1})	μ^- (cm^2 V^{-1} s^{-1})	n^+ (cm^{-2})	n^- (cm^{-2})
Dark	0.23	0.23	4.3×10^{11}	1.0×10^{11}
Illuminated	16.98	0.21	3.6×10^{11}	1.1×10^{11}

The transport measurement data reveal an asymmetry in electron/hole dynamics in the dark and under illumination. Despite the substantial increase in mobility, a slight reduction in hole density upon illumination suggests that photogenerated holes are efficiently extracted. However, this could also point to fast recombination processes at the interface, as inferred from the quenching of PL in thick CrSBr/MoS_2 samples.

In contrast, the electron dynamics follows a different trend: electron mobility decreases slightly under illumination, while the electron density remains stable. This behavior indicates both the efficient charge separation at the heterojunction and suppression of electron transport, possibly due to trapping at the semiconductor interface or defect states.[18–20] This is rationalized by shifting the V_{th} to more negative values under illumination, suggesting that trapped electrons generate a local electric field that counters the external gate voltage.[21] Furthermore, the trapped electrons may

scatter free electrons, leading to a reduction in electron mobility.

Based on the thorough investigation of the heterostructures in various configurations (vertical and lateral junctions) and the consistent experimental outcomes, we rule out environmental doping effects[22,23]as the primary factor influencing the device's optoelectronic performance. Instead, we attribute the observed behavior to interface-driven phenomena between the two materials.

S9. Frequency- and Amplitude-Dependent AC Response of the Device

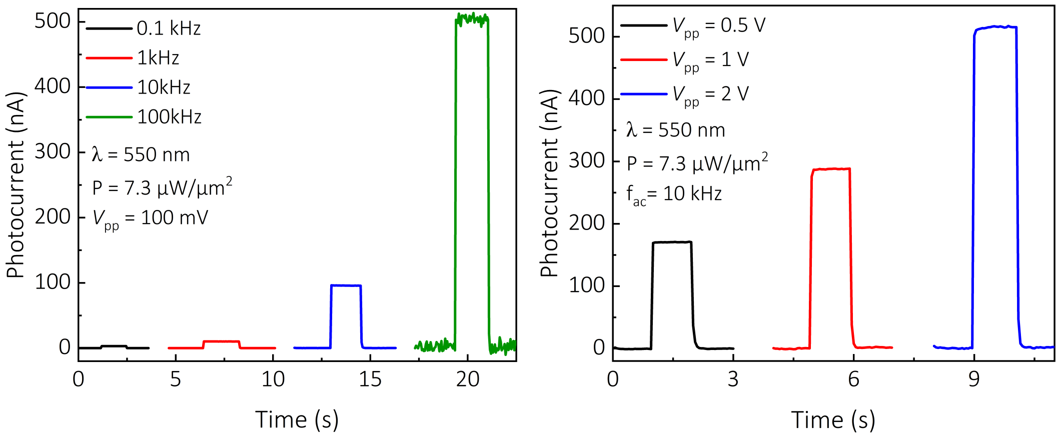

Figure S12: Photocurrent changes with frequency (left panel) and amplitude (right panel) of the AC signal in a thick CrSBr/MoS_2 device.

The AC regime enhances the charge carrier extraction by a periodic alteration of the electric field direction at higher frequencies, helping to overcome potential barriers caused by the band offsets. Figure S12 illustrates how the alternating voltage's increasing amplitude and frequency effectively lower these barriers, which are more restrictive under DC conditions.

As the AC frequency increases, the rapid oscillations of the electric field effectively smooth out the potential barriers, causing their effective height to fluctuate. Instead of facing a static barrier under DC conditions, the holes encounter a dynamic and oscillating potential landscape.[24,25] This variation in the barrier height makes it easier for the charge carriers to traverse the junction in a time-averaged manner, facilitating increased transport across the interface.[26]

As the AC voltage amplitude increases, the alternating electric field strengthens, further enhancing the effective electric field across the heterostructure. A stronger field causes a more pronounced

modulation of the energy barriers, leading to their effective transient flattening. This dynamic modulation allows holes to gain enough energy to surmount the barrier momentarily. Both these parameters enhance hole conduction by reducing the impact of the band offsets and improving extraction efficiency.

S10. Amplitude-Dependent Capacitance Change

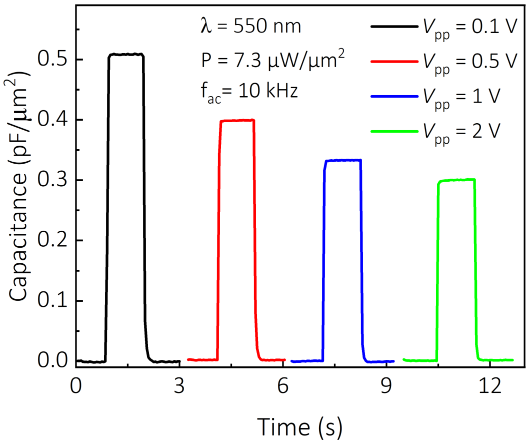

Figure S13: Variation in the capacitance of the CrSBr/MoS_2 device with interrupted illumination.

To further investigate the photoresponse dynamics of the device under AC conditions, we followed capacitance (C) changes during interrupted illumination and variable amplitude of the driving voltage. Under the typical conditions used in this study (f_{AC} = 10 kHz, V_{pp} = 0.1 V), we observed a 0.5 pF/μm^2 increase in C during the illumination of the heterojunction (Figure S13). An increase in C indicates the accumulation of photogenerated carriers at the CrSBr/MoS_2 interface and possibly at the electrode/material interface due to the Schottky barrier contact. The photogenerated holes and electrons widen the depletion region, raising the system's capacitance.

As the amplitude of the applied AC voltage increases, a decrease in C is observed. This behavior aligns with the known relationship between capacitance and voltage in semiconductor junctions, where the capacitance decreases as the applied voltage increases. At higher AC amplitudes, the oscillating electric field depletes charge carriers at a higher rate, reducing the net accumulation of charges at the junction. This narrows the depletion width, consequently lowering the overall C.

This dynamic modulation suggests a trade-off between the enhanced carrier extraction and the diminishing efficiency of charge accumulation as the amplitude of the AC voltage increases. While the illumination-induced carrier generation initially increases C, higher voltage amplitudes reduce the system's ability to store charges and increase the number of charge carriers available for extraction and transport.

The increase in AC capacitance upon illumination, contrasted with the reduction in hole density observed in the transfer characteristics, reflects two distinct carrier dynamics. The increased capacitance suggests the transient accumulation of photogenerated carriers at the junction or electrode/material interface. In contrast, the reduced hole density indicates a depletion of the majority carrier population under steady-state conditions.

S11. Summary of AC Characteristics of the Device

Finally, we describe a complex interplay between the most critical AC properties of the thick CrSBr/MoS_2 lateral junction device under study (Figure S14). The top panel shows the frequency dependence of the capacitance and dielectric loss measured using the loss of tangent (tan δ, also commonly referred to as a loss factor or dissipation factor). We observe similar behavior in the dark and under illumination, with corresponding shifts attributed to the photoinduced carrier generation.

At low frequencies, photoexcited charges are effectively stored at the junction, resulting in high C. However, as the frequency rises, these charges struggle to follow the rapid oscillations of the AC field, causing a drop in C. The loss factor peaks at 1/2 of the capacitance drop and is related to the maximum energy dissipation (tan δ = 0.3 at 5 Hz (dark) and 86 Hz (illuminated), corresponding to time constants of 200 ms and 12 ms, respectively). The tan δ typically peaks near relaxation or resonance frequencies, associated with material-specific polarization mechanisms such as interface dipole relaxation, defect states polarization (leading to charge trapping/de-trapping), or recombination.[27] In our case, we rule out recombination losses due to the significant difference in time scales (picosecond decay seen in TRPL measurements). Instead, we attribute the observed dielectric loss to the interface polarization within the heterostructure. Here, the applied electric field

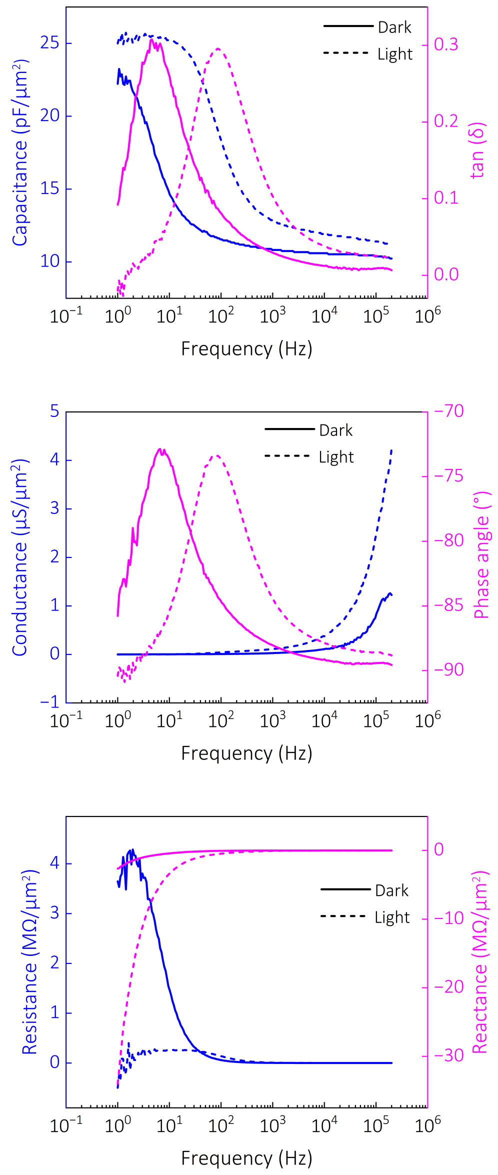

Figure S14: AC frequency dependence of selected properties of thick $CrSBr/MoS_2$ lateral junction device collected in the dark and under illumination. Capacitance and loss factor (top panel), conductance and phase angle (middle panel), resistance and reactance (bottom panel).

interacts strongly with the interfacial dipoles, delaying their response to the field and resulting in energy dissipation, typically manifested as material heating.

These observations align with the phase angle maximum of –73° (middle panel) appearing at the same frequency as tan δ. This indicates that the system is not purely capacitive, which would correspond to a phase angle of –90°, but has significant resistive or dissipative components. The

fact that both the phase angle and tan δ peaks are observed simultaneously reinforces the notion that the system undergoes frequency-dependent charge-relaxation processes. As the frequency increases, charge carriers can no longer follow the oscillating electric field, resulting in a reduction of the phase shift, reaching an almost purely capacitive response (–90°). However, this decay toward a lower plateau in C (~ 11 pF/μm^2) at higher frequencies implies that the system transitions from a carrier-induced capacitance regime (significant charge-carriers contribution) to a geometrical capacitance-dominated regime, which is determined mainly by the physical structure of the device, such as the spacing between the electrodes and material dimensions and dielectric properties.

Our conclusions regarding interface polarization are further supported by the change in reactance (X, bottom panel), which is negative at lower frequencies and reaches zero at the same frequencies when the capacitance reaches a steady state lower plateau. Typically, negative X is associated with capacitive behavior in AC circuits, as the reactance of a capacitor is given by

$$X = -\frac{1}{2\pi f C}$$

where f is the frequency. The negative sign represents the phase shift introduced by the capacitor, where the voltage lags the current by 90°. When the reactance reaches zero, the system transitions between a reactive energy storage regime (negative X) and a resistive dissipation regime. Additionally, we observe a large change in reactance depending on illumination, which further reinforces the idea that this effect is due to photoinduced carrier dynamics.

Finally, when the C reaches a plateau and X approaches zero, there is a corresponding increase in AC conductance (G, middle panel) and a decrease in the AC resistance (R) in the dark (bottom panel). As the frequency rises, the time available for charge carriers to become trapped at the interface or defects is reduced, decreasing effective resistance. Notably, under illumination, a phenomenon resembling negative differential resistance is observed, similar to what was previously seen in DC measurements (Figure 3b, main text). This may be attributed to excessive charge accumulation or trapping at defect states, which generates a local electric field opposing the

external AC field, thereby creating an effective "reverse current" at low frequencies. An alternative explanation could involve band-to-band tunneling, though a comprehensive analysis of this effect would be needed.

The combined effects of C, tan δ, G, R, and X, provide a clear picture of how photoexcited charges behave under varying frequencies in the CrSBr/MoS_2 device. At low frequencies, the high C, negative X, and large tan δ values suggest that the charges primarily accumulate at the junction, undergoing significant energy dissipation due to accumulation/trapping and resistive processes (as seen by high R in the dark). The system behaves like a capacitor, where charges follow the field and are stored in the external circuit. However, at higher frequencies, the drop in C, zero X, and negligible tan δ, along with decreasing R and increasing G, indicates more efficient charge transport across the heterojunction. The frequency blurs potential barriers in a time-averaged manner, facilitating the transfer of charges across the junction rather than storing them, reducing energy dissipation and resistive losses.

S12. Photocurrent Independence of the Source/Drain Polarity

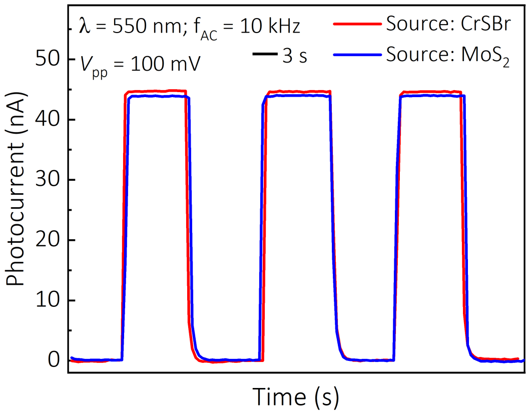

Figure S15: AC photocurrent in thick CrSBr/MoS_2 device with different source/drain polarity.

To further investigate the potential effects of the source/drain material on photocurrent behavior, we conducted additional AC photocurrent measurements using the opposite polarity of source/drain electrodes (Figure S15). Remarkably, these measurements consistently revealed a positive polarity of the AC photocurrent, regardless of whether CrSBr or MoS_2 was used as the source electrode.

S13. Detectivity and Gain

In order to quantify the AC photodetector's capability to detect weak optical signals and amplify the resulting photocurrent, we evaluate its performance using detectivity (D*) and gain (G):

$$D^* = \frac{RA}{2eI_{\textbf{dark}}}$$

where R is responsivity, I_{dark} is the dark current, and A is the active device area.

$$G = \frac{Rh\nu}{e\eta}$$

where h is the Planck constant, ν is the photon frequency, and $\eta = 1$ is the quantum efficiency.

We summarize these parameters in Figure S16. In a low-power regime using an 800 nm wavelength, we achieved detectivity of 1.5×10^{13} Jones and a corresponding gain amplification of 3.5×10^{5}, which places the thick CrSBr/MoS_2 heterostructure among top-tier photodetectors. This high detectivity arises from the combination of a large responsivity, a relatively low dark current, $I_{\text{dark}} = 9.15\times10^{-7}$ A, and low noise spectral density, $S_{\text{n}} = 1.7\times10^{-12}$ A/(Hz)$^{1/2}$ at 0.33 Hz. Additionally, the calculated gain agrees very well with the responsivity, suggesting efficient optoelectronic operation in the AC regime.

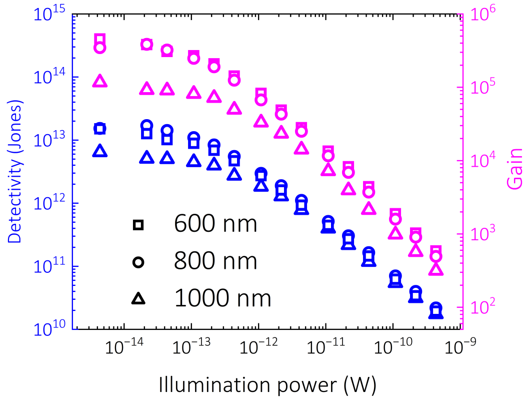

Figure S16: Detectivity and gain of MoS_2/CrSBr photodetector working in AC regime.

S14. Figure of Merit Comparison with the State-of-the-Art Systems

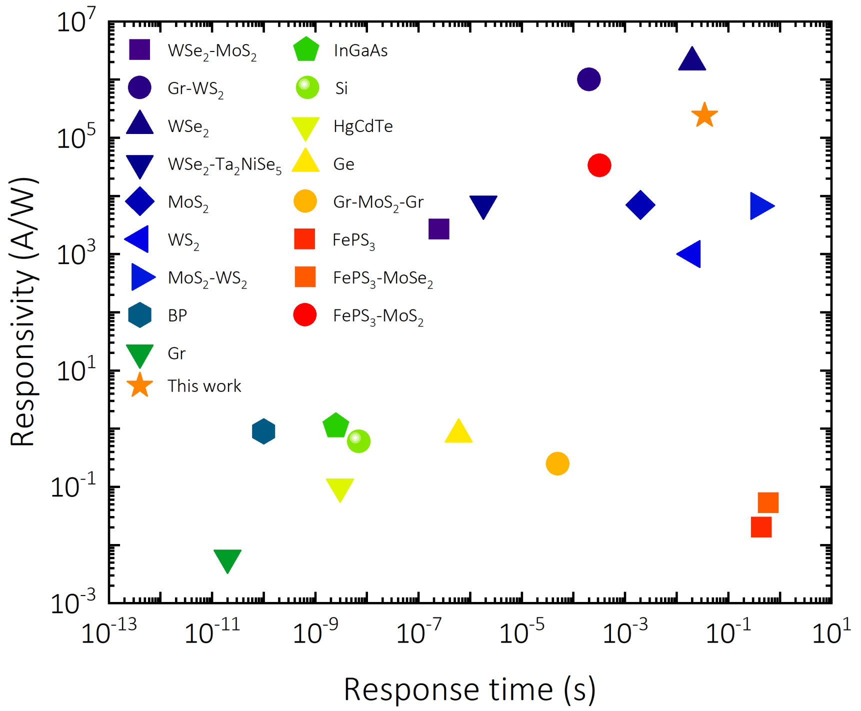

Figure S17: Comparison of the performance metrics in traditional and 2D-based photodetectors.[28–34]

S15. Photoluminescence of CrSBr

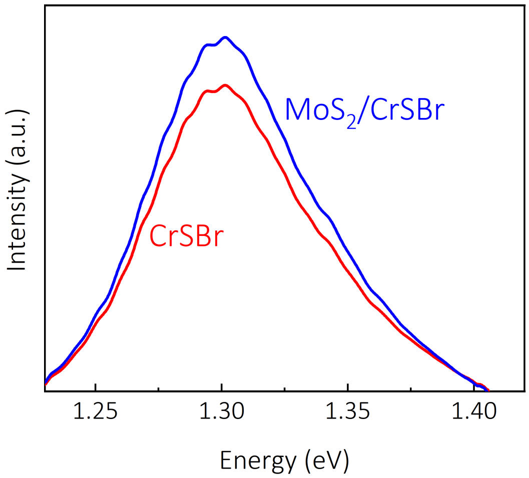

Figure S18: PL spectra of bare CrSBr and CrSBr underneath 1L MoS_2 detected using 2.33 eV laser excitation along the b-axis of CrSBr. Spectra were smoothened to dampen the oscillations produced due to the spectrometer's operation in the low-energy regime.

PL spectrum of CrSBr was measured using a 2.33 eV laser in the backscattering geometry. CrSBr exhibits a prominent emission centered around 1.3 eV (Figure S18), which is attributed to the radiative recombination of excitons near the direct band gap transition at the Γ point in the Brillouin zone.[1] The electronic band gap and the exciton binding energy in CrSBr have been estimated to be approximately 1.95 eV and 0.5 eV,[7,8,35] which is reflected well by the PL emission energy of 1.3 eV measured here.

S16. Raman Spectra of CrSBr

Raman spectroscopy using a 2.33 eV laser excitation was used to analyze the samples. To account for the crystal anisotropy of CrSBr, we aligned the excitation and detection along the a- or b-crystallographic axis of CrSBr. Figure S19 shows the Raman spectra of CrSBr, displaying three, previously reported modes, A_g^1, A_g^2, and A_g^3.[36–38] When the laser was polarized along the a-axis, we observe only the A_g^2 mode at 244.7 cm^{-1}. In contrast, with polarization along the b-axis, all three modes at frequencies of 113.6 cm^{-1}, 244.6 cm^{-1}, and 342.6 cm^{-1} are visible.[38]

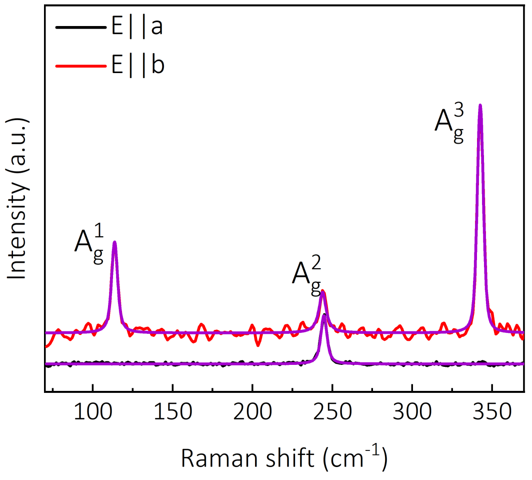

Figure S19: Raman spectra of 82 nm-thick CrSBr under 2.33 eV excitation, polarized along the a- and b-axis, respectively. Magenta curves are the fitted spectra.

S17. CrSBr Thickness-Dependent Raman Spectra of MoS_2

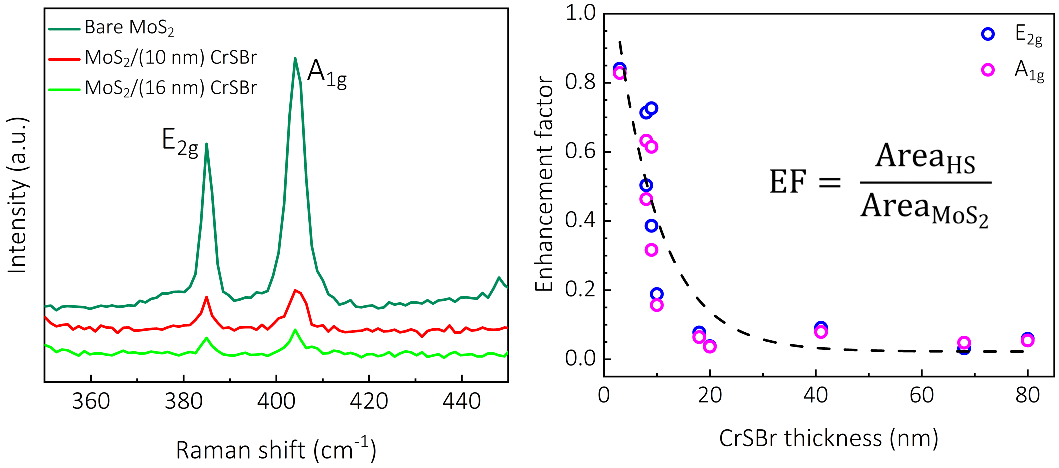

Figure S20: Left panel: Raman spectra of MoS_2 in the three heterostructure regions discussed in the main text, that is, bare MoS_2 on SiO_2/Si (teal), MoS_2/10 nm CrSBr (red), and MoS_2/16 nm CrSBr (green). MoS_2/41 nm CrSBr is not shown as the signal was close to the noise level. Right panel: MoS_2 Raman enhancement factor for E_{2g} and A_{1g} modes as a function of the CrSBr layer thickness. The dashed black curve is a guide to the eye. Raman enhancement factor was defined as the ratio of the heterostructure to bare MoS_2 peak areas, $EF = Area_{HS}/Area_{MoS_2}$.

The non-resonant Raman spectrum of bare MoS_2 consist of two main vibrational modes visible in Figure S20 (left panel) and Figure S21: E_{2g} at ~ 385 cm^{-1}, caused by the in-plane vibrations of two S atoms relative to the Mo atom, and A_{1g} at ~ 404 cm^{-1}, caused by the out-of-plane vibrations

Table S3: Fitted values of the Raman peak positions and full widths at half maximum (FWHM) of both E_{2g} and A_{1g} modes of MoS_2 for the spectra shown in Figure S20 (left panel).

	Position (cm^{-1})		FWHM (cm^{-1})	
	E_{2g}	A_{1g}	E_{2g}	A_{1g}
Bare MoS_2	385.2	404.3	2.5	3.9
MoS_2/(10 nm) CrSBr	384.8	404.5	2.4	3.6
MoS_2/(16 nm) CrSBr	384.9	404.2	2.0	2.7

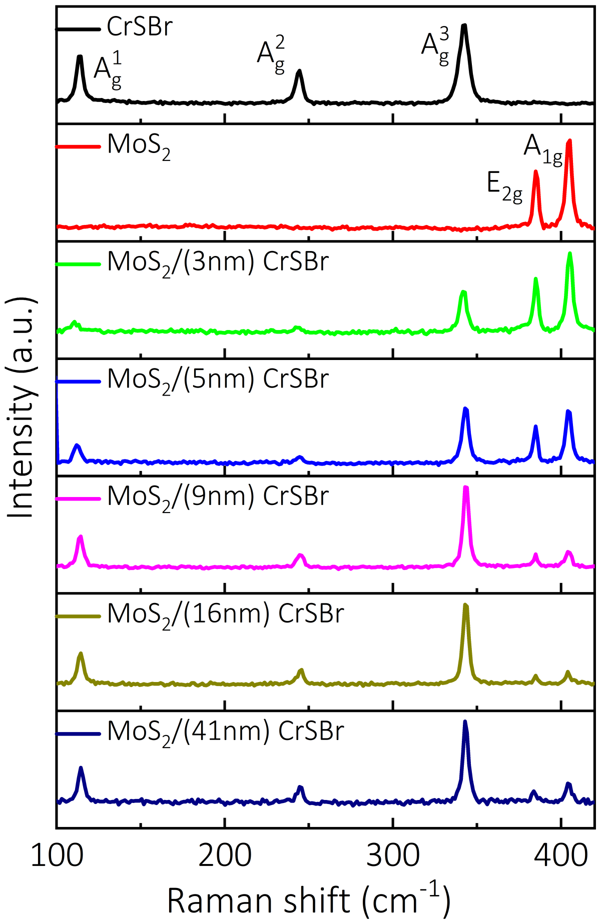

Figure S21: Raman spectra of CrSBr and MoS_2/x nm-thick CrSBr heterostructures (where x = 0, 3, 5, 9, 16, 41) under 2.32 eV laser excitation polarized along the b-axis of CrSBr.

of S atoms in the opposite direction. The frequency difference between these two peaks decreases with decreasing number of layers due to decreased dielectric screening and reaches approximately 19 cm^{-1} in monolayer MoS_2.[39,40]

For the heterostructures, two observations were made. First, there was a slight (<0.5 cm^{-1}) shift in the Raman shift frequencies for both modes (Table S3), possibly due to substrate-induced charge transfer and/or strain.[41] Second, the intensities of both modes were significantly reduced compared to that of MoS_2. To understand the quenching effect on the Raman intensities of MoS_2 on top of CrSBr, we measured the Raman spectra from all samples (which were also used for thickness-dependent PL in the main text) and plotted the enhancement factor (EF) as a function of the CrSBr thickness (Figure S20 right panel). The Raman EF follows the same trend as that of the PL EF (Figure 1f in the main text), which is expected since both MoS_2 vibrational modes couple with the A^0 exciton.[42]

Surprisingly, despite the significant enhancement in the PL, we observed no enhancement of the Raman intensities. This suggests that an opposing phenomenon may be causing the reduction in the intensity of MoS_2 Raman for all CrSBr thicknesses. It also indicates that without the presence of this counter effect, the PL EF values would likely be even higher due to exciton-phonon coupling in MoS_2.[42]

S18. Interference Effect

The thickness of the underlying substrate or another 2D material plays an important role in affecting the interference of both absorption and emission of light.[43,44] Therefore, it is necessary to consider the possibility of such effects here.

To that end, we prepared new samples in the inverted geometry, that is, CrSBr on top of MoS_2, and performed the PL and Raman measurements. Figure S22 shows the PL and Raman spectra for different thicknesses of CrSBr in this inverted geometry. The PL intensity is clearly strongly enhanced for the thinnest CrSBr ($\sim$ 7 nm). Upon increasing the thickness of CrSBr, the PL intensity is reduced and eventually quenched for a 27-nm-thick CrSBr flake. On the other hand, the

Raman intensities of both modes are suppressed for all CrSBr thicknesses, in accordance with our measurements for MoS_2 on top of CrSBr flakes. These observations confirm that the interference effects play little role in the observed PL intensity changes and that the charge transfer drives the variation in PL at the interface.

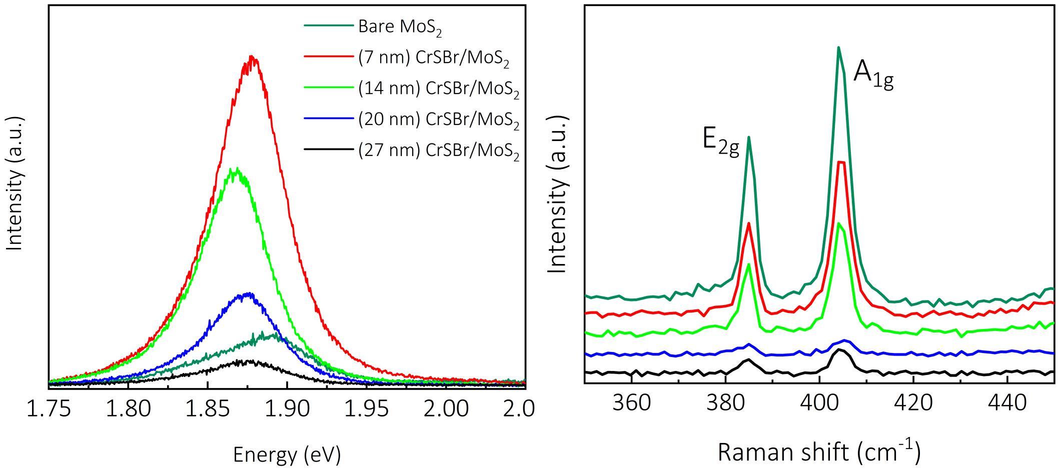

Figure S22: PL (left panel) and Raman (right panel) spectra of the inverted heterostructure samples, i.e. CrSBr on top of MoS_2.

S19. Environmental Effect

Several studies report on the PL enhancement of MoS_2 due to the defect passivation by the strongly-binding atmospheric oxygen.[45,46] Since all our samples were prepared in the air, it was essential to assess the role of oxygen and/or other molecules in the enhancement and quenching of the PL of MoS_2 in contact with CrSBr. Thus, we assembled another sample inside the controlled environment of a glovebox to avoid trapping oxygen, water, and other organic molecules and characterized it in the air for comparison with the air-assembled samples. Figure S23 shows the PL and Raman spectra of bare MoS_2 and MoS_2/6 nm CrSBr heterostructure prepared in this manner. Once again, we observed the PL enhancement and Raman quenching when the MoS_2 flake was on top of the CrSBr flake.

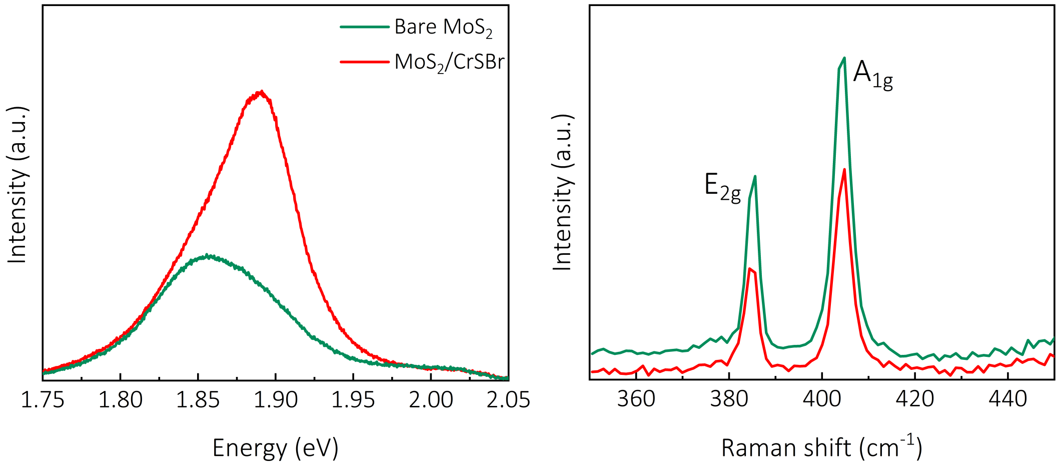

Figure S23: PL (left panel) and Raman (right panel) spectra of the samples prepared inside a controlled environment of a glovebox.

S20. Current-Voltage and Transfer Characteristics of another Vertical Junction Device

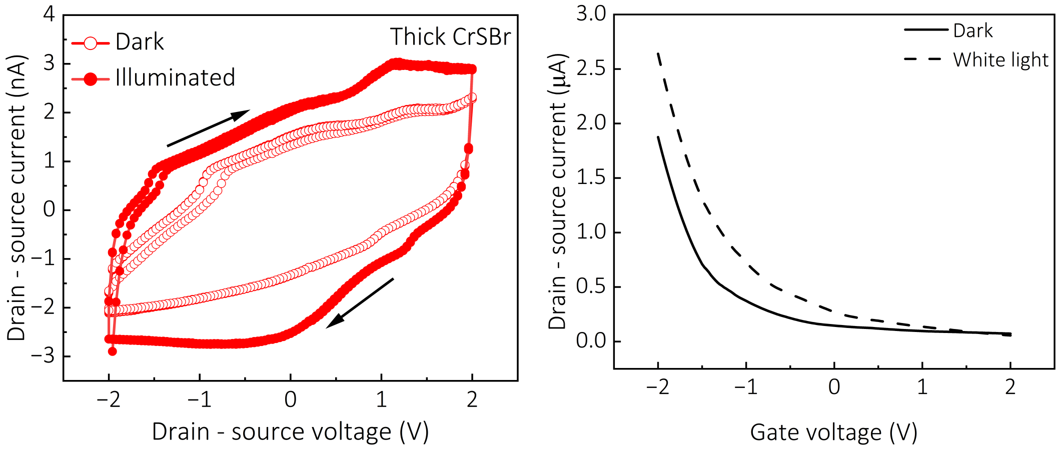

Figure S24: Current-voltage characteristics (left panel) of thick CrSBr/MoS_2 vertical heterojunction in the dark (open circles) and under illumination (filled circles). Transfer characteristics (right panel) of the same device in the dark (solid line) and under illumination (dashed line) show consistent p-type behavior when in contact with thick CrSBr.

References

1. Lin, K.; Sun, X.; Dirnberger, F.; Li, Y.; Qu, J.; Wen, P.; Sofer, Z.; Söll, A.; Winnerl, S.; Helm, M.; others Strong Exciton–Phonon Coupling as a Fingerprint of Magnetic Ordering in van der Waals Layered CrSBr. *ACS Nano* **2024**, *18*, 2898–2905.

2. Ryou, J.; Kim, Y.-S.; Kc, S.; Cho, K. Monolayer MoS_2 Bandgap Modulation by Dielectric Environments and Tunable Bandgap Transistors. *Scientific Reports* **2016**, *6*, 29184.

3. Bianchi, M.; Hsieh, K.; Porat, E. J.; Dirnberger, F.; Klein, J.; Mosina, K.; Sofer, Z.; Rudenko, A. N.; Katsnelson, M. I.; Chen, Y. P.; others Charge Transfer Induced Lifshitz Transition and Magnetic Symmetry Breaking in Ultrathin CrSBr Crystals. *Physical Review B* **2023**, *108*, 195410.

4. Vaquero, D.; Clericò, V.; Salvador-Sánchez, J.; Martín-Ramos, A.; Díaz, E.; Domínguez-Adame, F.; Meziani, Y. M.; Diez, E.; Quereda, J. Excitons, Trions and Rydberg States in Monolayer MoS_2 Revealed by Low-temperature Photocurrent Spectroscopy. *Communications Physics* **2020**, *3*, 194.

5. Zhu, J.; Wang, Z.; Yu, H.; Li, N.; Zhang, J.; Meng, J.; Liao, M.; Zhao, J.; Lu, X.; Du, L.; others Argon Plasma Induced Phase Transition in Monolayer MoS_2. *Journal of the American Chemical Society* **2017**, *139*, 10216–10219.

6. Ramos, M.; Marques-Moros, F.; Esteras, D. L.; Mañas-Valero, S.; Henríquez-Guerra, E.; Gadea, M.; Baldoví, J. J.; Canet-Ferrer, J.; Coronado, E.; Calvo, M. R. Photoluminescence Enhancement by Band Alignment Engineering in MoS_2/$FePS_3$ van der Waals Heterostructures. *ACS Applied Materials & Interfaces* **2022**, *14*, 33482–33490.

7. Watson, M. D.; Acharya, S.; Nunn, J. E.; Nagireddy, L.; Pashov, D.; Rosner, M.; van Schilfgaarde, M.; Wilson, N. R.; Cacho, C. Giant Exchange Splitting in the Electronic Structure of A-type 2D Antiferromagnet CrSBr. *npj 2D Materials and Applications* **2024**, *8*, 54.

8. Smolenski, S.; Wen, M.; Li, Q.; Downey, E.; Alfrey, A.; Liu, W.; Kondusamy, A. L.; Bostwick, A.; Jozwiak, C.; Rotenberg, E.; others Large Exciton Binding Energy in a Bulk van der Waals Magnet from Quasi-1D Electronic Localization. *Nature Communications* **2025**, *16*, 1134.

9. Fernández Garrillo, P. A.; Grévin, B.; Chevalier, N.; Borowik, Ł. Calibrated Work Function Mapping by Kelvin Probe Force Microscopy. *Review of Scientific Instruments* **2018**, *89*, 043702.

10. Shakya, J.; Kumar, S.; Kanjilal, D.; Mohanty, T. Work Function Modulation of Molybdenum Disulfide Nanosheets by Introducing Systematic Lattice Strain. *Sci. Rep.* **2017**, *7*, 9576.

11. Lattyak, C.; Gehrke, K.; Vehse, M. Layer-Thickness-Dependent Work Function of MoS_2 on Metal and Metal Oxide Substrates. *J. Phys. Chem. C* **2022**, *126*, 13929–13935.

12. Lee, S. Y.; Kim, U. J.; Chung, J.; Nam, H.; Jeong, H. Y.; Han, G. H.; Kim, H.; Oh, H. M.; Lee, H.; Kim, H.; Roh, Y.-G.; Kim, J.; Hwang, S. W.; Park, Y.; Lee, Y. H. Large Work Function Modulation of Monolayer MoS_2 by Ambient Gases. *ACS Nano* **2016**, *10*, 6100–6107.

13. Mondal, M.; Manchanda, P.; Saha, S.; Jangid, A.; Singh, A. Quantification of Two-Dimensional Interfaces: Quality of Heterostructures and What Is Inside a Nanobubble. *ACS Applied Materials & Interfaces* **2024**, *16*, 42608–42614.

14. Golovynskyi, S.; Datsenko, O. I.; Dong, D.; Lin, Y.; Irfan, I.; Li, B.; Lin, D.; Qu, J. Trion Binding Energy Variation on Photoluminescence Excitation Energy and Power During Direct to Indirect Bandgap Crossover in Monolayer and Few-layer MoS_2. *The Journal of Physical Chemistry C* **2021**, *125*, 17806–17819.

15. Hu, L.; Liu, X.; Dalgleish, S.; Matsushita, M. M.; Yoshikawa, H.; Awaga, K. Organic Optoelectronic Interfaces with Anomalous Transient Photocurrent. *Journal of Materials Chemistry C* **2015**, *3*, 5122–5135.

16. Pulikodan, V. K.; Raees A, M.; Alexander, A.; Nalledath, A. K.; Namboothiry, M. A. Origin of

Anomalous Transient Photocurrent in Solution-Processed WS_2 Nanosheet-Based Self-Powered Photodetectors. *ACS Applied Nano Materials* **2024**, *7*, 8007–8021.

17. Ortiz-Conde, A.; García-Sánchez, F. J.; Muci, J.; Barrios, A. T.; Liou, J. J.; Ho, C.-S. Revisiting MOSFET Threshold Voltage Extraction Methods. *Microelectronics Reliability* **2013**, *53*, 90–104.

18. Furchi, M. M.; Polyushkin, D. K.; Pospischil, A.; Mueller, T. Mechanisms of Photoconductivity in Atomically Thin MoS_2. *Nano Letters* **2014**, *14*, 6165–6170.

19. Zhao, Y.; Xiong, T.; Liu, Y.-Y.; Jiang, X. Reliability Improvement of 2-D WSe_2 FETs by Regulating Charge Trapping: An Ab Initio Demonstration. *IEEE Transactions on Electron Devices* **2024**, *71*, 6410–6416.

20. Shockley, W.; Read Jr, W. Statistics of the Recombinations of Holes and Electrons. *Physical Review* **1952**, *87*, 835.

21. Yu, W. J.; Liu, Y.; Zhou, H.; Yin, A.; Li, Z.; Huang, Y.; Duan, X. Highly Efficient Gate-tunable Photocurrent Generation in Vertical Heterostructures of Layered Materials. *Nature Nanotechnology* **2013**, *8*, 952–958.

22. Siao, M.; Shen, W.; Chen, R.; Chang, Z.; Shih, M.; Chiu, Y.; Cheng, C.-M. Two-dimensional Electronic Transport and Surface Electron Accumulation in MoS_2. *Nature Communications* **2018**, *9*, 1442.

23. Park, Y.; Li, N.; Jung, D.; Singh, L. T.; Baik, J.; Lee, E.; Oh, D.; Kim, Y. D.; Lee, J. Y.; Woo, J.; others Unveiling the Origin of n-type Doping of Natural MoS_2: Carbon. *npj 2D Materials and Applications* **2023**, *7*, 60.

24. Büttiker, M.; Thomas, H.; Prêtre, A. Current Partition in Multiprobe Conductors in the Presence of Slowly Oscillating External Potentials. *Zeitschrift Für Physik B Condensed Matter* **1994**, *94*, 133–137.

25. Zhou, X.-F.; Zhao, X.-A.; Zheng, J.-L. The Instability of Internal Potential in Double-barrier Structure. *International Journal of Modern Physics B* **2008**, *22*, 5103–5109.

26. Kobayashi, N.; Masumoto, H.; Takahashi, S.; Maekawa, S. Giant Dielectric and Magnetoelectric Responses in Insulating Nanogranular Films at Room Temperature. *Nature Communications* **2014**, *5*, 4417.

27. Wang, X.; Zhang, W.; Ji, X.; Zhang, B.; Yu, M.; Zhang, W.; Liu, J. 2D MoS_2/graphene Composites with Excellent Full Ku Band Microwave Absorption. *RSC Advances* **2016**, *6*, 106187–106193.

28. Konstantatos, G. Current Status and Technological Prospect of Photodetectors Based on Two-Dimensional Materials. *Nature Communications* **2018**, *9*, 5266.

29. Liu, M.; Wei, J.; Qi, L.; An, J.; Liu, X.; Li, Y.; Shi, Z.; Li, D.; Novoselov, K. S.; Qiu, C.-W.; others Photogating-assisted Tunneling Boosts the Responsivity and Speed of heterogeneous WSe_2/Ta_2NiSe_5 Photodetectors. *Nature Communications* **2024**, *15*, 141.

30. Shin, G. H.; Park, C.; Lee, K. J.; Jin, H. J.; Choi, S.-Y. Ultrasensitive Phototransistor Based on WSe_2–MoS_2 van der Waals Heterojunction. *Nano Letters* **2020**, *20*, 5741–5748.

31. Ye, K.; Liu, L.; Liu, Y.; Nie, A.; Zhai, K.; Xiang, J.; Wang, B.; Wen, F.; Mu, C.; Zhao, Z.; others Lateral Bilayer MoS_2–WS_2 Heterostructure Photodetectors with High Responsivity and Detectivity. *Advanced Optical Materials* **2019**, *7*, 1900815.

32. Duan, J.; Chava, P.; Ghorbani-Asl, M.; Lu, Y.; Erb, D.; Hu, L.; Echresh, A.; Rebohle, L.; Erbe, A.; Krasheninnikov, A. V.; others Self-driven Broadband Photodetectors Based on $MoSe_2$/$FePS_3$ van der Waals n–p Type-II Heterostructures. *ACS Applied Materials & Interfaces* **2022**, *14*, 11927–11936.

33. Ramos, M.; Carrascoso, F.; Frisenda, R.; Gant, P.; Mañas-Valero, S.; Esteras, D. L.; Bal-

doví, J. J.; Coronado, E.; Castellanos-Gomez, A.; Calvo, M. R. Ultra-broad Spectral Photoresponse in $FePS_3$ Air-stable Devices. *npj 2D Materials and Applications* **2021**, *5*, 19.

34. Long, M.; Shen, Z.; Wang, R.; Dong, Q.; Liu, Z.; Hu, X.; Hou, J.; Lu, Y.; Wang, F.; Zhao, D.; others Ultrasensitive Solar-Blind Ultraviolet Photodetector Based on $FePSe_3/MoS_2$ Heterostructure Response to 10.6 μm. *Advanced Functional Materials* **2022**, *32*, 2204230.

35. Wilson, N. P.; Lee, K.; Cenker, J.; Xie, K.; Dismukes, A. H.; Telford, E. J.; Fonseca, J.; Sivakumar, S.; Dean, C.; Cao, T.; others Interlayer Electronic Coupling on Demand in a 2D Magnetic Semiconductor. *Nature Materials* **2021**, *20*, 1657–1662.

36. Cenker, J.; Sivakumar, S.; Xie, K.; Miller, A.; Thijssen, P.; Liu, Z.; Dismukes, A.; Fonseca, J.; Anderson, E.; Zhu, X.; others Reversible Strain-induced Magnetic Phase Transition in a van der Waals Magnet. *Nature Nanotechnology* **2022**, *17*, 256–261.

37. Lee, K.; Dismukes, A. H.; Telford, E. J.; Wiscons, R. A.; Wang, J.; Xu, X.; Nuckolls, C.; Dean, C. R.; Roy, X.; Zhu, X. Magnetic Order and Symmetry in the 2D Semiconductor CrSBr. *Nano Letters* **2021**, *21*, 3511–3517.

38. Torres, K.; Kuc, A.; Maschio, L.; Pham, T.; Reidy, K.; Dekanovsky, L.; Sofer, Z.; Ross, F. M.; Klein, J. Probing Defects and Spin-Phonon Coupling in CrSBr via Resonant Raman Scattering. *Advanced Functional Materials* **2023**, *33*, 2211366.

39. Zhou, K.-G.; Withers, F.; Cao, Y.; Hu, S.; Yu, G.; Casiraghi, C. Raman Modes of MoS_2 used as Fingerprint of van der Waals Interactions in 2-D Crystal-based Heterostructures. *ACS Nano* **2014**, *8*, 9914–9924.

40. Lee, C.; Yan, H.; Brus, L. E.; Heinz, T. F.; Hone, J.; Ryu, S. Anomalous Lattice Vibrations of Single-and Few-layer MoS_2. *ACS Nano* **2010**, *4*, 2695–2700.

41. Chae, W. H.; Cain, J. D.; Hanson, E. D.; Murthy, A. A.; Dravid, V. P. Substrate-induced Strain

and Charge Doping in CVD-grown Monolayer MoS_2. *Applied Physics Letters* **2017**, *111*, 143106.

42. Qian, C.; Villafañe, V.; Petrić, M. M.; Soubelet, P.; Stier, A. V.; Finley, J. J. Coupling of MoS_2 Excitons with Lattice Phonons and Cavity Vibrational Phonons in Hybrid Nanobeam Cavities. *Physical Review Letters* **2023**, *130*, 126901.

43. Zhang, H.; Wan, Y.; Ma, Y.; Wang, W.; Wang, Y.; Dai, L. Interference Effect on Optical Signals of Monolayer MoS_2. *Applied Physics Letters* **2015**, *107*, 101904.

44. Quan, S.; Wang, Y.; Liang, Y.; Jiang, J.; Zhong, B.; Yu, K.; Zhang, H.; Kan, G. Interference Effect on Photoluminescence Intensity in GaSe up to 200 Layers. *The Journal of Physical Chemistry C* **2020**, *124*, 10185–10191.

45. Nan, H.; Wang, Z.; Wang, W.; Liang, Z.; Lu, Y.; Chen, Q.; He, D.; Tan, P.; Miao, F.; Wang, X.; others Strong Photoluminescence Enhancement of MoS_2 Through Defect Engineering and Oxygen Bonding. *ACS Nano* **2014**, *8*, 5738–5745.

46. Wang, W.; Shu, H.; Wang, J.; Cheng, Y.; Liang, P.; Chen, X. Defect Passivation and Photoluminescence Enhancement of Monolayer MoS_2 Crystals Through Sodium Halide-assisted Chemical Vapor Deposition Growth. *ACS Applied Materials & Interfaces* **2020**, *12*, 9563–9571.
